# Kraft (Nano)Lignin as Reactive Additive in Epoxy Polymer Bio-Composites

**DOI:** 10.3390/polym16040553

**Published:** 2024-02-18

**Authors:** Christina P. Pappa, Simone Cailotto, Matteo Gigli, Claudia Crestini, Konstantinos S. Triantafyllidis

**Affiliations:** 1 Department of Chemistry, Aristotle University of Thessaloniki, 54124 Thessaloniki, Greece; christipappa@chem.auth.gr; 2 Department of Molecular Sciences and Nanosystems, Ca’ Foscari University of Venice, 30170 Venice Mestre, Italyclaudia.crestini@unive.it (C.C.); 3 Center for Interdisciplinary Research and Innovation (CIRI-AUTH), 57001 Thessaloniki, Greece

**Keywords:** lignin–epoxy composites, kraft lignin, nanolignin, bio-composites, epoxy resins

## Abstract

The demand for high-performance bio-based materials towards achieving more sustainable manufacturing and circular economy models is growing significantly. Kraft lignin (KL) is an abundant and highly functional aromatic/phenolic biopolymer, being the main side product of the pulp and paper industry, as well as of the more recent 2nd generation biorefineries. In this study, KL was incorporated into a glassy epoxy system based on the diglycidyl ether of bisphenol A (DGEBA) and an amine curing agent (Jeffamine D-230), being utilized as partial replacement of the curing agent and the DGEBA prepolymer or as a reactive additive. A D-230 replacement by pristine (unmodified) KL of up to 14 wt.% was achieved while KL–epoxy composites with up to 30 wt.% KL exhibited similar thermo-mechanical properties and substantially enhanced antioxidant properties compared to the neat epoxy polymer. Additionally, the effect of the KL particle size was investigated. Ball-milled kraft lignin (BMKL, 10 μm) and nano-lignin (NLH, 220 nm) were, respectively, obtained after ball milling and ultrasonication and were studied as additives in the same epoxy system. Significantly improved dispersion and thermo-mechanical properties were obtained, mainly with nano-lignin, which exhibited fully transparent lignin–epoxy composites with higher tensile strength, storage modulus and glass transition temperature, even at 30 wt.% loadings. Lastly, KL lignin was glycidylized (GKL) and utilized as a bio-based epoxy prepolymer, achieving up to 38 wt.% replacement of fossil-based DGEBA. The GKL composites exhibited improved thermo-mechanical properties and transparency. All lignins were extensively characterized using NMR, TGA, GPC, and DLS techniques to correlate and justify the epoxy polymer characterization results.

## 1. Introduction

The worldwide dwindling of fossil resources, along with the environmental impact of their intensive and unsustainable use to produce fuels, chemicals, and materials, has created the need to search for alternative, eco-friendly, and renewable resources [1,2,3]. Thermoset and thermoplastic polymers are some of the most commonly used classes of materials in everyday to high-tech applications and their production had been traditionally based on petroleum and coal-derived chemicals [4]. Furthermore, most of these synthetic polymers, such as polyolefins or epoxy-based resins and polycarbonates, exhibit high resistance to natural or chemical degradation, thus requiring extremely challenging and energy-intensive processes to recycle them within the concept of the circular economy [5,6,7]. Thus, over the last 2–3 decades, the research and development focus has shifted towards the development of advanced green and sustainable biorefining processes for bio-based platform chemicals, monomers, and polymers, in addition to bio-fuels [8,9].

Lignocellulosic biomass has tremendous potential in this effort. More specifically, the three main structural components of lignocellulosic biomass, i.e., cellulose, hemicellulose, and lignin, represent an abundant source of chemicals that can be derived from chemo- and bio-catalytic depolymerization and transformations of the above biopolymers [2,10,11,12]. To this end, lignin is considered as the most abundant natural source of phenolic and aromatic compounds with huge valorization and exploitation potential [13,14,15,16]. Lignin is a highly crosslinked natural aromatic polymer, with more than 100 million tons of annual production, mainly in the form of lignosulphonates and kraft lignin as by-products of the pulp and paper industry and the rest originating from alkaline pulping, enzymatic hydrolysis, and organosolv pretreatment of biomass [17,18]. Despite its valorization potential, lignin is underutilized and is mainly used as solid biofuel for in-house energy and heating demands. Lignin’s macromolecule consists mainly of phenylpropanoid monomers, namely, coniferyl (G units), sinapyl (S units), and p-coumaryl (H units) alcohols with various functional groups, such as hydroxyl, carbonyl, methoxy, and carboxyl groups [19,20,21]. In the last decades, due to its structure and hydroxylated surface, lignin has attracted increased scientific and industrial interest in fields like energy, fuels, chemicals, and, more recently, in the polymer science field, aiming towards its utilization either with or without functionalization for the production of new green polymers and composites. So far, lignin has been employed in the synthesis of various thermosetting and thermoplastic polymers [13,22,23], while more recently, many studies have focused on lignin being utilized in resins, adhesives, and polymer blends [24,25,26,27,28,29,30,31]. Lignin has been successfully studied in the synthesis of epoxy resins replacing, at least partially, petroleum-derived phenolic prepolymers and curing agents [32]. Due to its phenolic/aromatic structure, lignin exhibits high chemical similarity and affinity with phenol-based epoxy resins, in addition to being prone to chemical functionalization via glycidylation (or other reactions) of its abundant hydroxyls [33,34,35,36,37].

Epoxy resins are considered one of the most commonly used thermosetting polymers in various applications because of their unique properties and performance towards chemical resistance, thermal stability, and adhesion strength to various substrates [38,39]. Epoxy resin is cured using various types of curing agents under different conditions depending on its specific application [40]. Currently, epoxy resins are produced by the reaction of the petroleum-based chemicals bisphenol-A (BPA) and epichlorohydrin that yield diglycidyl ether of bisphenol A (DGEBA) [41]. The major drawbacks of DGEBA-based resins are their limited recyclability [42] and the toxicity of bisphenol A (BPA) [43]. Therefore, there is an increasing interest in developing bio-based epoxy monomers or oligomers that can partly or completely replace petroleum-derived BPA monomers towards high-performance hybrid thermosets and composites.

Currently, studies of lignin–epoxy composites have focused on utilizing raw lignin as an additive in epoxy resin composites [36,44,45,46] or as a component capable of replacing either the epoxy resin prepolymer after chemical modification (glycidylation) [33,34,35,47] or the curing agent [46,48,49]. Relevant studies on lignin fractionation towards the achievement of better homogeneity, functionality, and polydispersity of lignins are also available [37,50,51,52,53,54,55,56,57,58,59]. Synthesis of thermosets like epoxy resins using unmodified lignins as additives–fillers represent a challenging field. Lignin, and in particular kraft lignin, is known for its condensed structure, resulting in poor solubility and dispersion in polymers. In our previous work, we demonstrated that sub-micro-sized organosolv beechwood lignin can be effectively incorporated in epoxy polymer composites without any modification [36]. In this work, we examined ways to overcome the challenge of kraft lignin’s dispersion and affinity with the epoxy matrix either by reducing its particle size or by chemically modifying lignin’s surface. Yin et al. reported the blending of lignin, epoxy resin, and polyamide in various loading levels, up to 70%, with successful crosslinking of the epoxy resin [60]. Sasaki et al. [61] prepared epoxy resins cured using the initial bamboo lignin. Kalili et al. [49] has also used lignin obtained from EFB black liquor as a curing agent with success in increasing the strength of the epoxy resin composite. Similar results were reported by Kong et al. [46] on the effect of the curing process of epoxy composites, attributed to the presence of hydroxyl groups of lignin that can promote the curing process.

To further enhance the miscibility and interaction of lignin with both the epoxy resin prepolymer and the curing agent, research has focused on the functionalization of the highly reactive lignin surface and its hydroxyl groups via glycidylation or amination reactions, respectively. The amination of lignin was achieved using the Mannich reaction [62,63] and was used to cure the epoxy resin. Modification of lignin with amine groups indeed increased the dispersion of lignin in the epoxy matrix [64] and its thermal stability [65]. However, in most cases, lignin is used as a prepolymer for epoxy composites due to its phenolic structure. Typically, lignin is glycidylized with epichlorohydrin to produce glycidyl phenolic oligomers in the presence of a phase transfer salt, such as tetramethylammonium hydroxide [61,66], or organic solvent mediums [33,47,67] and is being cured using various commercial aliphatic of aromatic curing agents [33,34,47,68]. Plenty of studies were successful in achieving fully cured lignin–epoxy composites, utilizing glycidylized lignins as the epoxy prepolymer [32,33,35,47,48,49,68,69,70,71,72,73]. Furthermore, alternative ways of achieving better dispersion into the polymeric matrixes need to be exploited, utilizing cost-effective and sustainable methods. The decrease of lignin’s particle size to achieve nano or micro-sized particles and its utilization into polymers is a field that has yet to gain extensive interest. Several methods have been already reported to obtain lignin-based nanoparticles, depending on the desirable size [36,52,74,75,76,77,78]. Among various strategies, ultrasonication of lignin suspensions has been one the most promising since it creates stable colloidal solutions of nano-sized particles that can be utilized towards improved dispersion and reinforcement in polymers and composites [52,79].

In this work, the synthesis of lignin–epoxy resin composites using softwood kraft lignin was demonstrated. More specifically, the effect of the as-received (pristine) kraft lignin as a curing agent as well as a reactive additive in commercial epoxy resins was studied in detail. To further enhance the reactivity and miscibility of kraft lignin with the epoxy–curing agent matrix, the parent lignin was functionalized with epoxy rings through glycidylation or, alternatively, it was subjected to ball milling or ultrasonication to reduce its particle size. The impact of lignin incorporation on the mechanical, thermal, and antioxidant properties of the lignin–epoxy resin composites was investigated.

## 2. Materials and Methods

### 2.1. Materials

Commercially available kraft lignin (370959, Sigma–Aldrich, St. Louis, MO, USA) (KL) was used after drying in a vacuum oven at 40 °C overnight. Epichlorohydrin (ECH), acetone, ethanol, and sodium hydroxide were all of analytical grade and were purchased from Sigma–Aldrich. The commercially available liquid epoxy resin used was a diglycidyl ether of bisphenol A (DGEBA, EPON 828, Hexion, Columbus, OH, USA) with an average epoxide equivalent weight EEW of 188.5 g/eq, as determined by titration (the producer provided range was 185–192 g/eq) and with Mw = 340 g/mol. The curing agent used was the aliphatic polyoxypropylene α,ω-diamine Jeffamine D-230 (AHEW = 60 g/eq, Mw = 230 g/mol, *n* = 2.6) (Huntsman Corporation, The Woodlands, TX, USA).

### 2.2. Treatment of Lignin

#### 2.2.1. Ball Milling of Initial Kraft Lignin (BMKL)

Ball milling was conducted on a Retsch S100 (Retsch GmbH, Haan, Germany) instrument. Approximately, 5 g of pre-dried kraft lignin was placed in an agate jar (80 mL) containing 8 balls (agate) with a diameter of 1 cm. Lignin was subjected to milling for a total duration of 8 h at 400 rpm (BMKL). The obtained lignin was dried in a vacuum oven at 40 °C overnight.

#### 2.2.2. Ultrasonication of Kraft Lignin towards Nano-Lignin (NLH)

Ultrasonication was performed using a Vibra-Cell sonicator (Model VC250, 20 KHz, 250 W) from Sonics & Materials, Inc. (Newtown, CT, USA), in pulse mode, equipped with a 1.2 cm diameter probe. A suspension of lignin in water was prepared at a concentration of 2% *w*/*v* and was left to stir at room temperature for 1 h, followed by 2 h of sonication, centrifugation (3000 rpm, 10 min), and drying under vacuum at 40 °C for 24 h.

#### 2.2.3. Synthesis of Glycidylized Lignin (GKL)

Glycidylation of the pristine kraft lignin was carried out without any previous solvent fractionation, depolymerization, or utilization of selected solvent-soluble fractions. The glycidylation process involved the following steps: In a round bottom flask, pristine kraft lignin (6 g) was dissolved in ECH (16 eq. of the number of active -OHs of the lignin as determined by ^31^P-NMR, see below) and acetone (200 mL). The mixture was stirred for 5 min at room temperature followed by the addition of aq. NaOH solution (3 eq. NaOH of the number of active -OHs of lignin) (200 mL). The mixture was stirred for 5 h at 55 °C. After the reaction, the mixture was cooled and iced H_2_O (400 mL) was added, followed by acidification to pH = 3 using a 1 M HCl solution and left overnight at 4 °C. The residue that formed at the bottom of the beaker was decanted in Falcon tubes (50 mL), iced H_2_O was added, and it was then centrifuged (3800 rpm, 20 min). The solid residue was further washed with iced H_2_O to remove any residual ECH. Finally, the recovered glycidylized lignin (GKL) was thoroughly dried at 40 °C for 24 h (yield = 96%).

### 2.3. Preparation of Lignin–Epoxy Composites

Neat pristine glassy epoxy polymer, used as control sample, was prepared by mixing the epoxy monomer DGEBA (EPON 828) with the curing agent (Jeffamine D-230) at 1:1 stoichiometric ratio of amine reactive hydrogens to epoxy rings, according to the respective epoxy (EEW) and amine-hydrogen equivalent weights (AHEW). The mixture was stirred at 55 °C for ~5 min followed by degassing (40 mbar, 10 min, 50 °C) to remove entrapped air and was finally cured in an oven (3 h at 75 °C and 3 h at 125 °C).

For the preparation of the lignin–epoxy composites, three approaches were applied. In the first approach, kraft lignin was used to partially replace the curing agent D-230, utilizing lignin’s OH groups (as determined by ^31^P-NMR, see below) as epoxy ring opening initiators. The replacement of the D-230 curing agent was in the range 5–14 wt.%. The prepared composites were coded as GL*x*/*y*KL, where *x* is the weight percent replacement of curing agent (5–14 wt.%) and *y* is the respective weight percent of kraft lignin in the composites (3–9 wt.%). In a typical experiment, KL lignin was dispersed in DGEBA, and the mixture was sonicated for 5 min followed by 1 h of stirring to evenly disperse the lignin (at 55 °C). D-230 curing agent was then added. The mixture was stirred for 5 min, degassed to remove entrapped air (40 mbar, 10 min, 50 °C), and was finally cured in an oven (3 h at 75 °C and 3 h at 125 °C).

In the second approach, GKL lignin was used as an epoxy prepolymer (i.e., replacing the commercial epoxy resin DGEBA). The prepared composites were coded as GL*x*/*yG*KL, where *x* is the weight percent replacement of epoxy resin (4–50 wt.%) and *y* is the respective weight percent of glycidylized lignin in the composites (3–40 wt.%). GKL lignin at selected amounts was dispersed in EtOH and sonicated for 15 min. The mixture was then poured into the liquid DGEBA and left to stir for 15 min to completely dissolve the GKL. The DGEBA/GKL mixture was sonicated for 3 extra min. The EtOH was evaporated at 60 °C and D-230 was added and stirred for 5 min. Degassing (40 mbar, 10 min, 50 °C) was applied to remove trapped air and solvent, and, finally, the mixture was cured in an oven (3 h at 75 °C and 3 h at 125 °C).

Finally, in the third route for the preparation of composites where lignin acted as an additive, dried lignin or lignin/EtOH suspensions were pre-mixed with the epoxy monomer EPON 828 at 60 °C for 1 h (evaporating the solvent when needed), followed by the addition and mixing with the curing agent for 5 min, degassing (40 mbar, 10 min, 50 °C), and finally curing in an oven (3 h at 75 °C and 3 h at 125 °C). More specifically, the stoichiometric molar ratio of commercial epoxy resin (DGEBA) and curing agent (Jeffamine D-230) was used and lignin was added in selected amounts, ranging from 1 to 45 wt.% of the DGEBA/D-230 mixture.

### 2.4. Characterization of Lignins and Lignin–Epoxy Composites

The elemental composition (C/H/N/S) of lignin was determined using an Elemental Analyzer (Eurovector EA3100 Series CHNS-O). Lignin samples were heated at 980 °C under a constant flow of helium (90 mL/min). The resulting gas products were analyzed using a gas chromatograph equipped with a thermal conductivity detector. The oxygen content was calculated by difference.

An LA-960 Laser Scattering Particle Size Distribution Analyzer (Horiba, Kyoto, Japan) was used to measure the particle size distribution of lignin samples. Aqueous lignin suspensions (2 *w*/*v*%) were prepared and shortly sonicated before analysis.

Molecular weight properties (weight- and number-average molecular weight (Mw, Mn) and polydispersity (PDI) of lignins were determined by GPC. The GPC analysis was performed using a Shimadzu Corporation instrument (Kyoto, Japan) consisting of a controller unit (CBM-20A), a pumping unit (LC-20AT), a degasser unit (DGU-20A_5R_), a column oven (CTO-20AC), a diode array detector (SPD-M20A), and a refractive index detector (RID-20A). Three analytical linear GPC columns (each 8 × 300 mm) were used for the analysis: Shodex KF-801, KF-802.5, and KF-803 (6 μm, with a Mw range of 100–70,000 Da). Molecular weights were given relative to polystyrene standards within the calibration range of 162–500,000 Da. Prior to the injection, all lignin samples were completely dissolved in THF (1–2 mg/mL) and filtered through a 0.45 μm PTFE syringe filter. A sample volume of 50 μL was injected. The analysis was performed using THF as the eluent with a constant flow rate of 0.5 mL/min, while the columns and the injection system were maintained at 42 °C.

The FT-IR-ATR analysis of lignins and composites was performed on an IR Tracer-100 (Shimadzu Corporation, Kyoto, Japan). Infrared transmittance spectra of lignin and composite powders were obtained in the range 450–4000 cm^−1^ at a resolution of 3 cm^−1^ using 32 co-added scans. All spectra presented are baseline corrected and normalized.

The thermogravimetric analysis (TGA) to determine the thermal stability and residual mass of lignins and lignin–epoxy composites was performed using a Netzsch STA 449F5 instrument (Netzsch Group, Selb, Germany). Pre-dried lignin samples or cured composites (5–10 mg) were placed in alumina crucibles and were heated under a 50 mL/min flow of N_2_ and a heating rate of 10 K/min in the temperature range of 25–950 °C (lignins) or 25–700 °C (composites).

A differential scanning calorimeter (DSC) analysis was performed in a Perkin–Elmer Pyris (Perkin Elmer Corporation, Waltham, MA, USA) for the determination of the glass transition temperature (T_g_) of the lignins and cured lignin-DGEBA mixtures. About 5–10 mg of pre-dried lignin samples was placed in a sealed aluminum crucible and heated from 20 °C to 105 °C at a heating rate of 10 °C, maintained for 20 min, followed by quenching at 20 °C, held at this temperature for 20 min, and finally heated to 200 °C at a heating rate of 10 °C/min. Regarding the lignin-DGEBA mixtures, approximately 5–10 mg was sealed in an aluminum crucible and subjected to a heating–cooling–heating cycle from 30–225 °C at 10 °C/min. During the analysis, an inert environment was maintained using nitrogen gas (50 mL/min).

The morphology of lignin samples was studied by scanning electron microscopy (SEM) using a JEOL JMS-840A instrument (JEOL Ltd., Akishima, Tokyo, Japan) operating at 20 kV equipped with an energy-dispersive X-ray spectroscopy (EDS) detector. Lignin powders samples were placed on double-sided carbon tape attached on an aluminum stub and were gold-sputtered to enhance conductivity.

High-resolution transmission electron microscopy (HRTEM) was performed using a JEOL 2011 microscope with an LaB6 filament, an accelerating voltage of 200 kV, a point resolution of 0.23 nm, and a spherical aberration coefficient of Cs = 1 mm. The lignin powder was placed onto a carbon lacey film supported on a 3 mm diameter and 300 mesh copper grid. The sample was further coated with a carbon layer to enhance its conductivity.

The porosity characteristics of all lignins were determined by N_2_ adsorption-desorption experiments performed at −196 °C in an Automatic Volumetric Sorption Analyzer (Autosorb-1 MP, Quantachrome, Boynton Beach, FL, USA). The samples were previously outgassed at 80 °C for 19 h under a 5 × 10^−9^ Torr vacuum. The BET area (total surface area) of the lignins was determined by the multi-point BET method and the total pore volume by the BJH analysis of adsorption data.

The NMR analysis was carried out in an Agilent 600 MHz spectrometer (Agilent Technologies, Santa Clara, CA, USA). Two-dimensional HSQC-NMR spectra were acquired using approximately 100 mg of the pre-dried lignin samples dissolved in 0.5 mL of DMSO-d6 (dimethylsulfoxide-d6, 99.8%, Deutero GmbH, Kastellaun, Germany). The chemical shifts were referenced to the solvent signal (2.50/39.52 ppm). The relaxation delay was set to 5 s, the number of scans was 16, and 450 increments (t1) were recorded in the ^13^C dimension. Prior to Fourier transformation, FID (free induction decay) signals were apodized with a *π*/2 sine squared–bell function in both dimensions. A baseline and manual phase correction were also applied to both dimensions. The relative abundance of the different lignin bonding types lignin was calculated by a semiquantitative integration of the respective correlation peaks. The area obtained from the integration of the *G*_2_ units was set as 100 aromatic units (*Ar*) and the amount of each linkage type (expressed as a number per 100 *Ar*) was calculated with the equation:

(1) $$I\left(x\right)=\frac{\int I(x)}{\int {I(G}_{2})}\times 100$$

where *I*(*x*) is the integral value of the α-position (C_α_–H_α_) of the A (β-O-4), B (β-β), and C (β-5) types of inter-unit linkages and *I* (*G*_2_) is the integral value of *G*_2_. NMR data were processed with MestReNova (Version 12.0.2-2091).

The quantitative ^31^P-NMR analysis was performed as previously reported [80]. In detail, an accurately weighted amount of 39–40 mg of pre-dried lignin sample was dissolved in 0.5 mL of a deuterated chloroform and anhydrous pyridine solution (1:1.6, *v*/*v*) (Solvent A), and 100 μL of a solution of chromium(III) 2,4-pentanedionate (5 mg/mL) as relaxation agent and N-hydroxy-5-norbornene-2,3-dicarboxylic acid imide (NHND, 18 mg/mL) as the internal standard (dissolved in Solvent A) was added. The solution was stirred for 30 min to let the lignin to fully dissolve. Then, 100 μL of 2-Chloro-4,4,5,5-tetramethyl-1,2,3-dioxaphospholane (TMDP) was added dropwise under stirring and the mixture was transferred to a 5 mm NMR tube and taken for measurement in an Agilent 500 MHz spectrometer (Agilent Technologies, Santa Clara, CA, USA). The spectra were acquired from 120–190 ppm, with 128 scans and a relaxation delay of 10 s. Prior to the quantification analysis, the sharp peak corresponding to the TMDP+H_2_O was shifted and assigned to 132.2 ppm. NMR data were processed with MestReNova (Version 14.0.2-26256).

The epoxy content of the glycidylized lignin was determined using ^1^H-NMR analysis. Approximately, 20 mg of pre-dried glycidylized lignin was dissolved in 0.6 g of DMSO-d6 and approximately 4 mg of 2,3,4,5,6-Pentafluoro-benzaldehyde (PFB) as internal standard. To acquire a detailed spectrum, 32 scans and a relaxation delay of 13 s were selected. For quantification, the area of the signal at 10.14 ppm corresponding to the aldehyde of the PFB was correlated to the area of the signal at 2.83 ppm, which corresponds to the H_1_ proton of the oxirane ring. The spectrum was auto-baseline corrected.

An Axio Lab.A1 light microscope (Carl Zeiss, Jena, Germany) equipped with a microscopy camera (AxioCam ERc 5s Rev.2) was used to capture the optical microscopy images of lignin–epoxy composites using ZEN 2012 lite software (Version 1.1.2.0).

Tensile tests to record the stress–strain curves were performed on an Instron 3344 dynamometer, in accordance with ASTM D638 using a crosshead speed of 5 mm/min. Dog-bone-shaped specimens were prepared with dimensions of 12 × 3 × 3 mm (length in the narrow region; width; thickness). The exact dimensions of specimens were measured before testing.

The thermo-mechanical properties (storage modulus, tanD) of the epoxy composites were measured on a Perkin Elmer Diamond DMA analyzer (Perkin Elmer Corporation, Waltham, MA, USA). The bending method was used with a frequency of 1 Hz and applied force of 500 mN. Heating of the specimens was performed at 3 °C/min in the temperature range of 25 °C to 110 °C. The specimens had rectangular shapes with dimensions of 50 × 13 × 2 mm. The exact dimensions of each sample were measured before the test.

The antioxidant activity (free radical scavenging) of the samples was determined by the 2,2-Diphenyil-1-picrylhydrazyl (DPPH) method. Approximately 50 mg of epoxy composites was placed with 3 mL of a 60 μM DPPH/EtOH solution in a glass vial. The stock (60 μM) DPPH/EtOH solution was used as a reference (control). The vials were stored in a dark space. To measure the antioxidant activity of composites, the absorbance of each solution was recorded at selected time intervals (1–8 h, 24 h, and 48 h) using a UV-VIS spectrometer at 517 nm. The antioxidant activity (*AA*) was calculated using the following equation:

(2) $$AA(\%)=100\times \frac{Absorbance\;of\;control-absorbance\;of\;solution}{Absorbance\;of\;control}$$

Weight uptake and resistance to EtOH of lignin–epoxy composites were tested by submersing pre-weighed composites in EtOH. The composites were weighed at selected time intervals (8, 24, and 48 h, up to 30 days) and the weight uptake was calculated based on the following equation:

(3) $$Weight\;Uptake(\%)=100\times \frac{Ws-Wi}{Wi}$$

where *w_i_* is the weight of composites prior the submersion (initial) (g) and *w_s_* is the weight of the submerged and dried composites (g).

## 3. Results and Discussion

### 3.1. Physicochemical Characterization of Initial and Modified Lignins

The lignins used in this work were the commercial pristine kraft lignin (KL) and its three modified variants, i.e., ball-milled lignin (BMKL), nano-lignin (NLH) and glycidylized (GKL) lignin, as described in the experimental section. All lignins were analyzed using GPC, N_2_ adsorption-desorption porosimetry, and elemental analysis to determine their molecular weight (Mw) and polydispersity (PDI), their total pore volume and surface area, and their elemental composition, respectively. The Mw data and GPC curves are given in Table 1 and Figure S1, respectively. Pristine softwood kraft lignin (KL) shows a Mw of 6500 g/mol and a relatively high PDI of 4.7, which is expected due to the relatively harsh conditions of the isolation process. During kraft pulping, cleavage and repolymerization reactions of lignin-based fragments take place, affecting the molecular weight and structure of the native lignin [16,81]. Kraft lignin is thus composed of two main fractions: the first one showing a higher molecular weight and a higher concentration of aliphatic hydroxyl groups, and the second possessing a highly condensed structure, lower molecular weight, and a higher abundance of phenolic OH groups. The large and non-uniform molecular weight and PDI of kraft lignin has been a point of issue, especially towards the production of polymers.

Various methods have been employed to counteract this un-uniformity using chemical functionalization [22], depolymerization [82,83], and sequential fractionation [50]. Alternatively, mechanical treatment methods, such as ball milling, have been used to reduce the particle size of lignin and eventually affect structural/chemical properties. Ball milling has been mostly used to assist with lignin isolation from biomass [84,85]. In the present study, the pristine kraft lignin was ball milled (BMKL) to primarily decrease its particle size (as discussed below). However, as can be seen in Table 1, the Mw of BMKL is lower and the PDI is narrower compared to those of the pristine lignin as a result of the treatment. Similarly, when KL was ultrasonicated, the resulting nano-lignin (NLH) showed a significant reduction of the Mw compared to the pristine KL, from 6500 to 3200 g/mol. The chemical treatment applied in this work was the functionalization of lignin’s hydroxyls with epoxide groups (via glycidylation) in order to induce reactivity towards amine curing. Glycidylation has been extensively studied in the last decade as a potential way of obtaining bio-based lignin–epoxy polymers [33,47,67,86]. In most published works, lignins had to be pre-treated to attain dissolution in the reaction solvent. Most common pre-treatments were sequential solvent fractionation [87,88] or depolymerization [89,90] approaches. In the present study, the combination of an acetone and water mixture was found to be an excellent solvent that resulted in the complete dissolution of kraft lignin, thus utilizing the entire kraft lignin sample for glycidylation. A reduction of the Mw was also observed for the GKL sample, although to a lesser extent compared to the other modified lignins (BMKL and NLH), while PDI was also decreased compared to the pristine KL.

The elemental analysis of the various lignin samples is also shown in Table 1. The contents of C, H, N, S, and O were typical for this type of lignin [21].

The chemical structure and variety of bonds present in the structure of the pristine (KL) and treated lignins can be identified from the FT-IR-ATR spectra (Figure S2). The characteristic absorption bands of the parent KL lignin appear with a broad band at 3410 cm^−1^ corresponding to OH stretching, while the bands at 2932 and 2840 cm^−1^ are assigned to C-H stretching in CH_2_ and CH_3_ [72,91]. Absorption bands at 1684–1730 cm^−1^ are attributed to C=O stretching in carboxyl and ester groups. Four bands associated with the skeleton C-H and C=C bonds of the aromatic ring are found at 1590, 1514, 1138, and 754 cm^−1^ [72,91,92,93], the bands at 1456 and 1423 cm^−1^ are assigned to methoxy C-H bonds [35,92], while the bands at 1268 cm^−1^ (G ring) and 1218 cm^−1^ correspond to the C-C, C-O, and C=O bonds of the aromatic rings [35,92,94]. The band at 1367 cm^−1^ represents the phenolic -OH groups [35], while the bands found at 1081 and 1025 cm^−1^ correspond to the ether C-O-C bond and C-H ring deformation [92,93]. Bands corresponding to aromatic C-H bond vibrations, 1025 cm^−1^, 857 cm^−1^, and 812 cm^−1^, are typically found in guaiacyl monomers. The characteristic bands of the pristine KL lignin are also present in the BMKL and NLH samples, indicating the structural similarity between the three lignins. The successful functionalization of pristine kraft lignin with epoxy groups (GKL lignin) via glycidylation of lignin’s hydroxyls is also confirmed by the new characteristic absorption (oxirane C-O-C stretching) bands at 915 cm^−1^, 857 cm^−1^, and 759 cm^−1^ [73] that are present in their spectra along with the disappearance of the characteristic band at 1367 cm^−1^ assigned to phenolic -OH.

**Figure 1:**
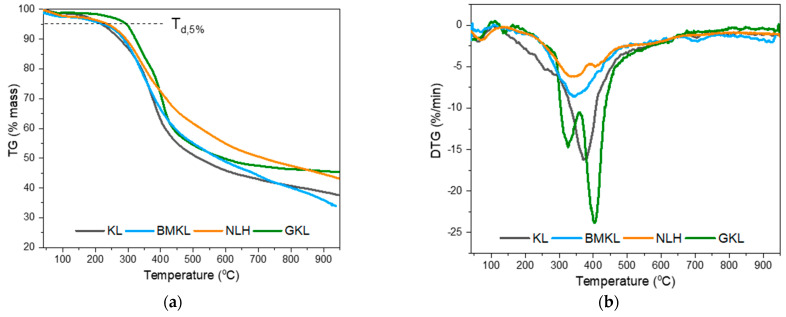
(**a**) TGA and (**b**) DTG curves of the different lignin samples (N_2_ atmosphere).

The thermal decomposition profile of the pristine and treated lignins are depicted in the thermogravimetric analysis (TGA) and differential thermogravimetric analysis (DTG) curves shown in Figure 1. The temperature at which 5% weight loss is observed (T_d,5%_) and the maximum degradation temperature (DTG_max_) are shown in Table 2. At lower temperatures (below 100 °C), only a slight weight loss was observed due to residual sorbed moisture release. KL, BMKL, and NLH lignins exhibit similar weight loss profiles with only one major step at temperatures of 170–500 °C and with a maximum (DTG) at about 340 to 372 °C. This steep weight loss is attributed to the thermal decomposition of the phenolic/aromatic polymeric structure of lignin. Furthermore, the relatively high residual mass at 800 °C (40–47%) is a confirmation of the high thermal stability of lignin’s aromatic nature [95], which is mainly converted into bio-char at those high temperatures. Overall, the thermal stability of kraft lignin was retained after the mechanical treatment with ball milling and ultrasonication (BMKL and NLH).

The thermal stability and degradation temperatures at 5% weight loss (T_d,5%_) of the GKL appear to be increased compared to the other samples. This could be attributed to the additional bonds and groups identified in the GKL sample, as well as to the cleavage of the initial β-O-4 ether bonds (as listed in Table 3). In addition, self-polymerization reactions might have taken place, leading to a higher degree of crosslinked network [96]. Furthermore, the glycidylized lignin exhibited weight loss in two steps (Figure 1b). The first step was observed in the range of 250–350 °C, and could be attributed to the breaking of the new ether bonds due to the addition of the epoxy rings or to the self-crosslinking of the glycidylized lignins via reactions between the epoxy groups and free hydroxyls, as heating of epoxy resins to a high temperature can promote homo-polymerization reactions [97]. The second mass loss peak observed at a higher temperature (>350 °C) can be associated with the degradation of initial lignin structure, which might have undergone patrial condensation during the glycidylation reaction [98]. The increase of the degradation temperature for glycidylized lignins compared to unmodified lignin has already been reported in the literature for kraft lignin [99].

Glass transition temperatures (T_g_) of lignins were determined by DSC analysis. The T_g_ of lignins can be affected by the molecular weight, degree of condensation (number of C-C linkages), as well as the content of phenolic OH [100,101]. KL and BMKL samples showed a relatively high, similar T_g_, while NLH and GKL exhibited a substantially lower T_g_ (Table 2), most likely due to lower amounts of intermolecular hydrogen bonds as well as a lower abundance of condensed structures like β-5, 5-5 and 4-O-5 [22] (Table 3).

**Figure 2:**
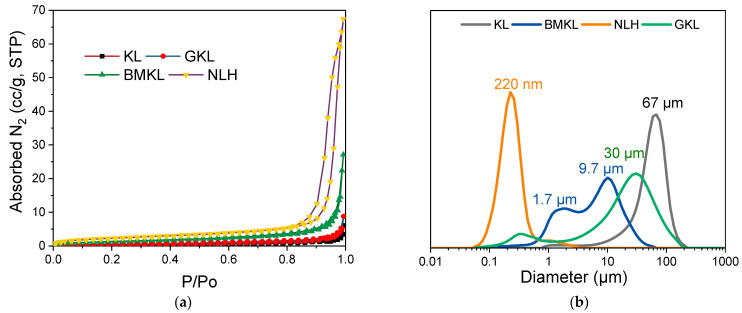
(**a**) Nitrogen adsorption–desorption isotherms and (**b**) particle size distribution (PSD) of lignins.

The total pore volumes and surface areas of KL, BMKL, and NLH were also investigated with N_2_ porosimetry; the respective N_2_ adsorption–desorption isotherms are shown in Figure 2. As listed in Table 1, the surface area and total pore volume of both BMKL and NLH increased significantly after treatment. In more detail, the surface area of BMKL lignin showed a 5-fold increase, while the NLH lignin showed a significant 11-fold increase compared to the initial KL. Following the same trend, the total pore volume of BMKL increased by 6-fold, while NLH presented also a significant 12-fold increase. The observed increase in the porous characteristics is attributed to the enhanced external surface area and the inter-(nano)particle voids formed upon the mechanical treatment of lignin with both mechanical methods, especially with ultrasonication, which resulted in smaller micro/nanoparticles (as discussed below).

**Figure 3:**
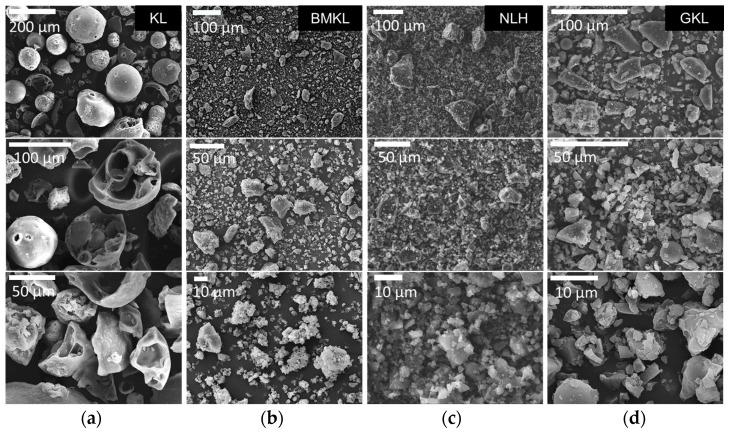
Morphological characterization by SEM of (**a**) pristine kraft lignin (KL), (**b**) ball-milled lignin (BMKL), (**c**) nano-lignin (NLH), and (**d**) glycidylized lignin (GKL).

**Figure 4:**
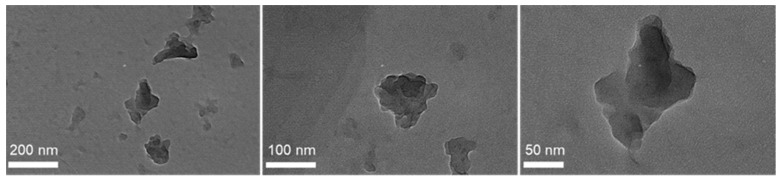
TEM images of NLH primary nanoparticles.

The particle size and morphology of all lignin samples were studied using laser scattering particle size distribution (PSD) analysis and scanning electron microscopy (SEM), respectively. As shown in Figure 3, KL particles are mostly characterized by monolithic and fragmented spherical particles of different sizes and diameters (50–200 μm). Based on the PSD analysis shown in Figure 2b, KL has a relatively narrow particle distribution with a maximum at ~67 μm. Regarding the modified lignins, their morphology is significantly altered. In particular, BMKL exhibits smaller primary particles of less than 10 μm in size, with few larger monolithic particles with size of up to ca. 50 μm. Accordingly, the PSD analysis of BMKL shows a bimodal particle size distribution with maxima at about 1.7 and 9.7 μm (Figure 2b). Similarly, the SEM images of the ultrasonicated sample (NLH) show significantly smaller primary particles compared to KL of ca. less than 5 μm in size, while the PSD analysis reveals a nano-sized distribution with an average size of 220 nm, as was also previously reported [52]. The nano-sized morphology is also confirmed by the TEM images shown in Figure 4, which show nanoparticles of 100–200 nm. Regarding the morphological characteristics of GKL, it is evident that after the glycidylation reaction, the initial spherical morphology of KL is lost and the sample is characterized by more irregular and monolithic-like particles with a variety of sizes, being mostly smaller than 50 μm. The average size from the PSD analysis of GKL is 30 μm (Figure 2b). The semiquantitative analysis of the samples via SEM/EDS (Figure S3) revealed the presence of expected elements (C, O, and S) found in kraft lignin similar to the one determined by elemental analysis (Table 1). Traces of additional elements such as sodium (Na) and chlorine (Cl) are attributed to the kraft pulping process and the glycidylation reaction, respectively.

The content of hydroxyl groups, i.e., aliphatic, phenolic and carboxyl groups, was determined by quantitative ^31^P-NMR analysis, and the results are listed in Table 3. The hydroxyl content of lignin is of particular interest in lignin–epoxy polymer production since it provides a way to assess their functionality, effectiveness, and incorporation in the polymeric network as crosslinkers. On the other hand, the semi-quantitative analysis of the main lignin motifs was obtained from HSQC analysis, and the data are summarized in Table 3, together with the peak assignment.

**Figure 5:**
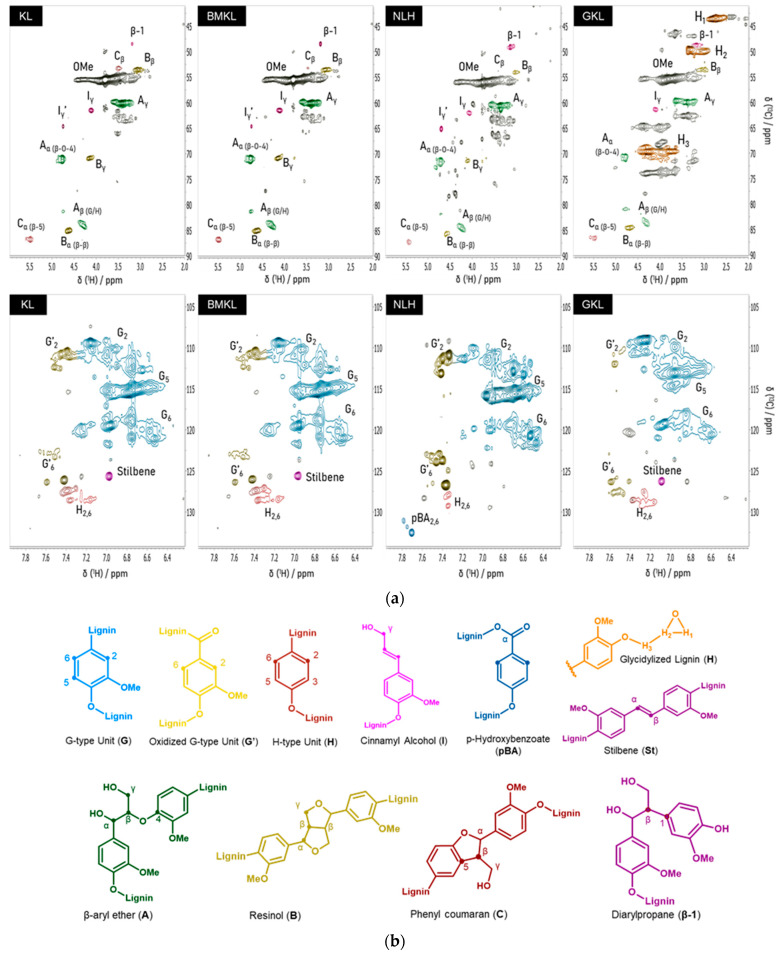
(**a**) Two-dimensional-HSQC NMR spectra of pristine kraft (KL), ball-milled lignin (BMKL), nano-lignin (NLH), and glycidylized lignin (GKL). The aliphatic region is shown in the first row, while the aromatic region is shown in the second. (**b**) Chemical structures of the main identified motifs present in the lignin samples.

Inter-unit linkages were quantified per 100 aromatic units (Ar) using the aromatic units (G_2_, C_2_/H_2_) as the internal standard. The KL lignin spectrum is shown in Figure 5a. Two-dimensional-HSQC NMR assignments of major structures in the spectra of lignins are listed in Table S1. As can be seen, the aromatic region of the spectrum is characterized by the strong guaiacyl (G units) signals at C_2_–H_2_ (δ_C_/δ_H_ 110–6.9 ppm), C_5_–H_5_ (δ_C_/δ_H_ 115.3/6.8 ppm), and C_6_–H_6_ (δ_C_/δ_H_ 119/6.8 ppm), and *p*-hydroxyphenyl (H units, C_2,6_-H_2,6_) signals (δ_C_/δ_H_ 128/7.2 ppm) [21]. On the other hand, in the oxygenated region, strong signals attributed to the methoxy groups (OMe, δ_C_/δ_H_ 55.6/3.7 ppm) are visible, together with the main inter-unit linkages. These are the β-O-4 (β-aryl ethers, type A linkage, 22.8 units per 100 Ar), β-β (pinoresinols, type B linkage, 10.9 units per 100 Ar), β-5 (phenylcoumarans, type C linkage, 6.9 units per 100 Ar) and β-1 (diarylpropane, 1.4 units per 100 Ar). The difference in the linkage content usually originates from both the type of biomass feedstock and the type of lignin isolation method [102]. Typically, β-O-4 linkages are the most abundant bonds in native lignins and the content of β-5, β-β and β-1 linkages is much lower [103]. Technical lignins, like kraft lignin, are expected to have a lower β-O-4 content compared to native lignins due to the harsh kraft pulping conditions, which induce specific structural alterations and ether bond cleavage [81,88]. Regarding the structure and OH content of the BMKL, no significant change is observed compared to the pristine lignin, testifying to its structural integrity after ball milling. In the case of the nano-lignin (NLH), in accordance with the data presented by Gilca et al. [52], ultrasound treatment resulted in an overall increase in the aliphatic OH content and carboxylic acids, most probably due to cleavage of aryl ether (β-O-4) bonds and lignin’s oxidation during the ultrasound treatment. On the other hand, the total phenolic OH content of NLH was similar to that of pristine KL (Table 3).

**Figure 6:**
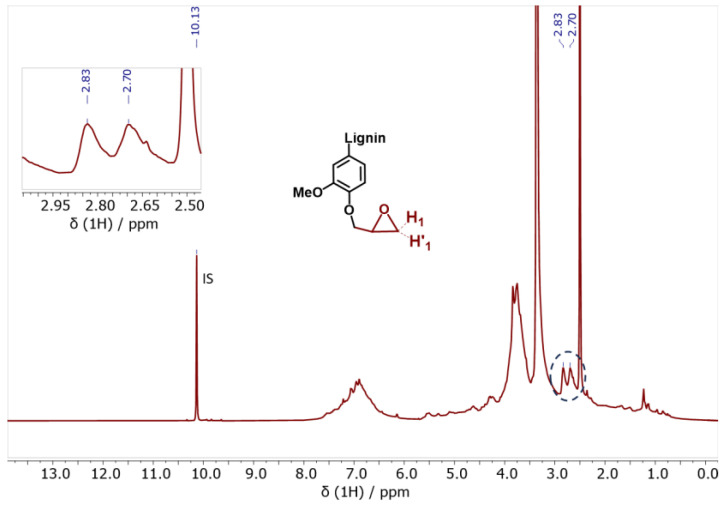
^1^H-NMR spectrum of the glycidylized kraft lignin (GKL). The characteristic epoxy ring peaks (H_1_ and H’_1_) are identified and quantified.

Regarding the glycidylized lignin, a significantly different behavior was observed. The content of phenolic OH and COOH groups dropped, indicating the success of the functionalization reaction with epoxy rings (Table 3 and Figure S4, Supporting Information). Overall, the degree of functionalization can be indirectly calculated based on the phenolic OH and COOH decrease and the contemporary increase in the aliphatic OH content. In fact, the aliphatic OH content was slightly increased, as already reported in the literature [88]. This is due to self-crosslinking and condensation reactions between the aliphatic OHs and the epoxy rings attached to the GKL. The free lignin -OH groups may indeed initiate a ring opening polymerization via nucleophilic addition that creates aliphatic -OHs. Alternatively, it may be due to undesired side reactions leading to oxirane ring opening, as shown in Scheme S1 (Supporting Information) and previously discussed [36]. The epoxy content has been also quantified using ^1^H-NMR (Figure 6). More specifically, it was determined by correlating the signal of the internal standard (IS) peak at 10.13 ppm to the H_1_ oxirane proton peak at 2.83 ppm and resulted in a value equal to 3.80 mmol epoxy rings/g, a value very close to the epoxide content calculated by ^31^P-NMR. The GKL epoxy content is translated to an epoxy equivalent weight (EEW) value, i.e., the weight in grams of resin that contain one mole equivalent of epoxide, of 263 g/eq. The determined EEW value of GKL is higher compared to that of the commercial DGEBA (~185–192 g/eq), but it still shows that GKL can be utilized as a DGEBA replacement, as shown below.

Regarding the overall structure of GKL lignin, the cleavage of major linkages like β-O-4, β-β, and β-5 is intensified after the glycidylation reaction (Table 3). The introduction of the epoxy rings on lignin’s structure is also qualitatively confirmed by the 2D-HSQC spectrum (Figure 5), where the peaks corresponding to the carbon atoms in the epoxy ring are clearly shown at δ_H_/δ_C_: 2.70 and 2.83/43.40 ppm (H_1_ and H_1_′), 3.29/49.50 ppm (H_2_), and 3.90 and 4.28/69.67 ppm (H_3_ and H_3_′) [33,67]. Moreover, the self-crosslinking of GKL that might occur to some degree can also be supported by the relevant 2D HSQC peaks in the GKL spectra (δ_H_/δ_C_: 73–74/4.2–4.4, 65/4.0–4.3 ppm).

### 3.2. Lignin–Epoxy Composites

#### 3.2.1. Reactivity of Kraft Lignin Ohs towards Epoxy Ring Opening

**Scheme 1:**
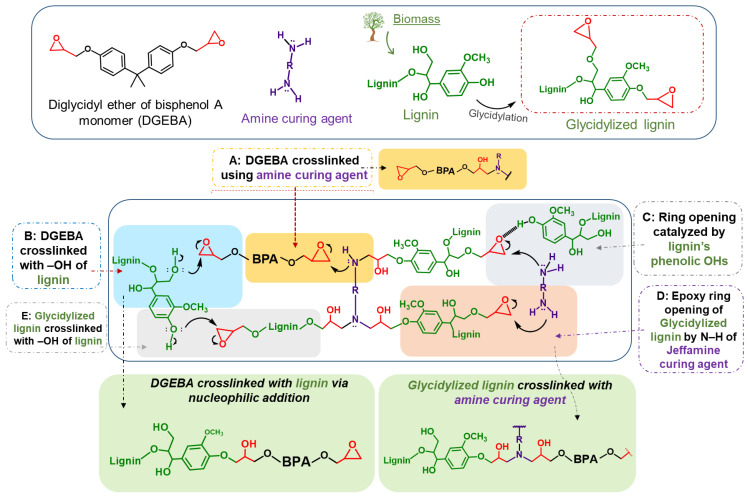
Proposed lignin/DGEBA/diamine crosslinking mechanism. Lignin can be utilized as a reactive additive or act both as a curing agent via its reactive Ohs or as an epoxy prepolymer via its epoxy rings (after glycidylation).

In general, the epoxy resin crosslinking mechanism is based on the opening of the DGEBA epoxide ring (-C-O-C) by the amine groups (-NH_2_) of the curing agents. The amine group reacts with the electrophilic carbon of the epoxide via nucleophilic addition, leading to C-O bond cleavage [104,105,106]. Apart from this typical “DGEBA and amine curing agent” crosslinking mechanism, the parent lignin and glycidylized lignin can also participate in the epoxy network expansion, as is shown in Scheme 1.

Kraft lignin, being a phenolic biopolymer, contains many reactive terminal hydroxyls and can be considered as a potential green and sustainable crosslinker (curing agent) of the epoxy polymer (DGEBA). In our previous work, we discussed the potential of organosolv lignin towards the ring opening of DGEBA’s epoxides via nucleophilic addition [36]. We suggested that lignin, through its abundant relatively acidic phenolic Ohs, can (i) participate in the crosslinking mechanism via the nucleophilic addition of the phenolic hydroxy group to the electrophilic carbon of the epoxide, leading to direct epoxy ring opening without an amine curing agent (Scheme 1, Method B, blue shading), as well as (ii) catalyze the DGEBA epoxy ring opening by amines, via the formation of hydrogen bonds between its Ohs and the DGEBA epoxy ring (Scheme 1, Method C, grey shading). Hydroxyl groups are present in lignin’s structure but are simultaneously the result of the epoxy ring opening, hence the (auto)catalytic nature of the reaction. In the case of glycidylized lignin (GKL), its epoxy rings can react with the amine curing agent (Scheme 1, Method D, orange shading) or even with lignin Ohs (Scheme 1, Method E, light grey shading), creating a strong network of interlinks. Therefore, the crosslinking mechanism of lignin (with or without glycidylation)/DGEBA/diamine mixtures is indeed complicated, multi-parametric and multi-influenced.

**Figure 7:**
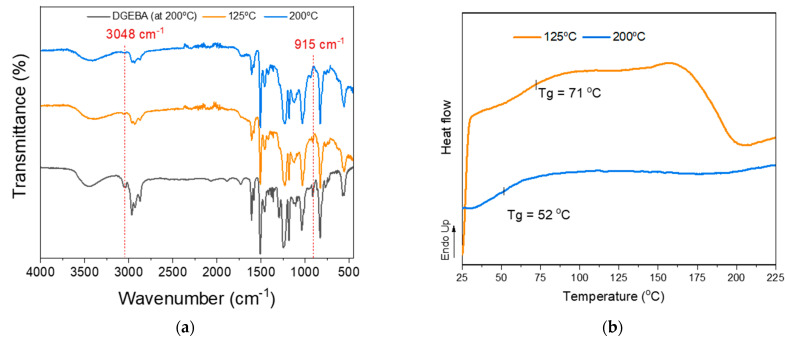
(**a**) FT-IR-ATR spectra and (**b**) DSC curves of pristine KL/DGEBA mixtures after curing stages: self-polymerization of DGEBA at 200 °C (black line), Stage 1—3 h at 75 °C followed by 3 h at 125 °C (orange line), and (Stage 2—3 h at 170 °C followed by 2 h at 200 °C (blue line).

Thus, we investigated more systematically the potential of utilizing kraft lignin (KL) as a curing agent. Mixtures of pristine kraft lignin (KL) with the commercial epoxy resin prepolymer EPON 828 (DGEBA) were prepared at an epoxy ring to lignin hydroxyl molar ratio of 1:1. The KL content in this formulation was 45 wt.%, while lignin’s OH content was determined by ^31^P-NMR analysis. The reactivity of KL towards ring opening and crosslinking with DGEBA (Scheme 1, Method B, blue shading) is highly related to the temperature applied. As can be seen in the FT-IR-ATR spectra and DSC curves (Figure 7a and b), the reactivity of KL towards the DGEBA epoxide requires a higher temperature compared to the Jeffamine D-230 curing agent due to the lower nucleophilicity of oxygen (of the -OH group) compared to nitrogen (of the -NH_2_ group) of the diamine. Figure 7a shows the FT-IR-ATR spectra of DGEBA and KL/DGEBA mixtures cured using different protocols. Initially, the self-polymerization of DGEBA resin at high temperatures was tested at 200 °C for 2 h (Figure 7a, black line). Then, the KL/DGEBA mixture was cured in two stages. The first stage included 3 h of heating at 75 °C followed by 3 h at 125 °C (Figure 7a, orange line), while the second (consecutive) stage included heating for 3 h at 170 °C followed by 2 h at 200 °C (Figure 7a, blue line). As can be seen from the spectra, DGEBA (after heating to 200 °C) displays characteristic peaks at 915 and 3048 cm^−1^ [107], typical of the epoxy rings, testifying to its stability and limited self-curing at 200 °C. Conversely, upon blending and reacting with KL at high temperature (200 °C), the DGEBA peaks at 915 and 3048 cm^−1^ disappear, while upon curing at lower temperature (125 °C), unreacted epoxy bands are still visible. The DSC analysis results (Figure 7b) support these findings, since the curing exotherm signal of the KL/DGEBA mixture disappeared upon curing at higher temperature. Therefore, the epoxy ring opening by lignin OH groups and the crosslinking of DGEBA with lignin as curing agent is possible, although at relatively higher temperatures.

#### 3.2.2. Dispersion of Kraft Lignin and Modified Lignins in Epoxy Composites

**Figure 8:**
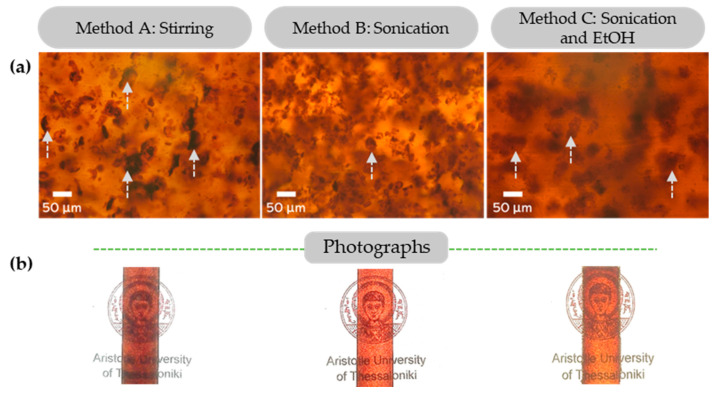
(**a**) Optical microscopy images of glassy lignin–epoxy composites containing 3 wt.% KL, prepared by Method A—stirring, Method B—sonication and Method C—combination of sonication and EtOH. (**b**) Respective photographs of the glassy lignin–epoxy composites (3 wt.% KL). Indicative lignin particles of increased size are indicated by arrows.

In our previous study [36], the high dispersion and almost complete solubilization of beechwood extracted sub-micro organosolv lignin in epoxy composites was demonstrated without the use of solvents, mechanical treatment or functionalization. However, the kraft lignin in this study, as most of the available technical lignins, does not exhibit the same ideal particle morphology/size properties. Despite this, the direct utilization of pristine KL as an epoxy polymer additive was tested. Three protocols for mixing the DGEBA prepolymer with the pristine lignin were investigated: (a) magnetic stirring (Method A), (b) sonication (Method B), and (c) dispersion in organic solvent (EtOH) and sonication (Method C). Glassy lignin–epoxy composites containing 3 wt.% KL were prepared with the abovementioned methods, and the respective photographs and optical microscope images are shown in Figure 8.

It is evident that both the sonication (Method B) and sonication/solvent (Method C) approaches induce a limited dissolution of the condensed lignin particles/agglomerates (dark, thick spots in the optical microscopy images) found in the stirred (Method A) sample, towards smaller and more dispersed particles.

**Figure 9:**
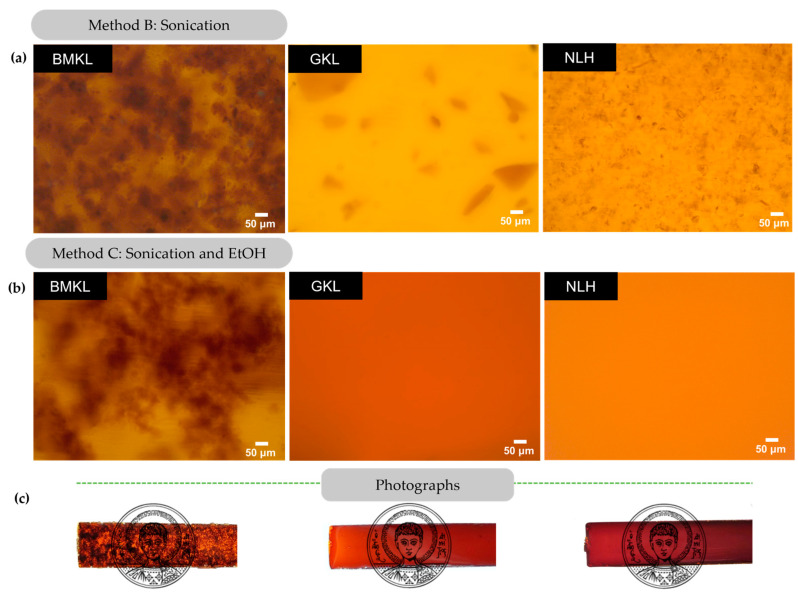
(**a**,**b**) Optical microscopy images of lignin–epoxy composites with 3 wt.% BMKL, GKL, and NLH lignins. The composites were prepared using sonication (Method B) or a combination of sonication and EtOH (Method C). (**c**) Photographs of the corresponding lignin–epoxy composites with 3 wt.% lignin.

The dispersion of the modified lignins within the composites was also studied using optical microscopy (Figure 9). It is clear that GKL and NLH lignins disperse almost completely just by sonication (Method B), in contrast to BMKL, as well as to the pristine KL (Figure 8). When EtOH was added (Method C), complete dispersion of GKL and NLH was observed and transparent epoxy–lignin composites were obtained (Figure 9). As can be seen in Figure S5, GKL and NLH lignins are not initially fully soluble in EtOH, while after sonication, both lignins swell. At this point, the suspension is poured into the liquid DGEBA and stirred. Within minutes, GKL and NLH lignins are fully solubilized in DGEBA and remain dissolved even after the removal of EtOH and addition of the D-230 curing agent. More importantly, the homogeneous dispersion and transparency are preserved even at higher lignin loadings (9–30 wt.%) (Figure S6). On the contrary, composites containing BMKL lignin (prepared with either of the three methods) are characterized by visible BMKL particles and aggregates are still present (Figure 9).

#### 3.2.3. Kraft Lignin as a Curing Agent—Thermo-Mechanical Properties

As previously mentioned, KL was studied as a curing agent, aiming towards the replacement of up to 100 wt.% of commercial curing agents. The prepared KL/DGEBA composites were very brittle due to the high mass ratio of lignin in DGEBA and thus were unable to be mechanically tested (the lignin content was as high as 45 wt.%). Subsequently, a commercial α, ω-polyoxypropylene diamine curing agent (Jeffamine D-230) was introduced to the mixture, as described in the Experimental Section. The amount of epoxy resin was fixed, while the amount of KL was gradually reduced, counterbalancing the hydroxyl reduction by the -NH_2_ groups of the Jeffamine D-230. A stoichiometric ratio of [epoxy rings]:[lignin’s Ohs + amine reactive hydrogens] of [1:1] was always maintained.

**Figure 10:**
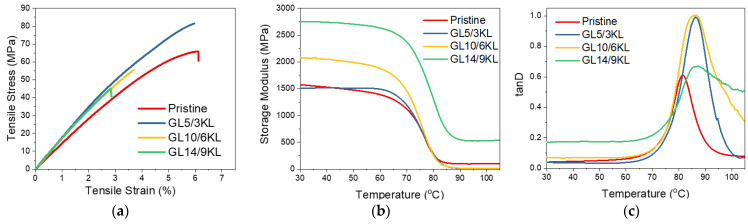
(**a**) Stress–strain curves, (**b**) storage modulus E’ curves, and (**c**) tanD curves of pristine KL–epoxy composites. KL was used as curing agent, replacing 5 wt.% of D-230 (3 wt.% KL content in the composite), 10 wt.% of D-230 (6 wt.% KL content), and 14 wt.% of D-230 (9 wt.% KL content).

Counterbalancing the amount of KL with Jeffamine D-230 and thus reducing the total lignin content (from 45 wt.%) resulted in less brittle composites. Tensile stress–strain curves of the composites containing KL up to and 9 wt.% can be seen in Figure 10. Composites with 3 wt.% KL, which corresponds to a 5 wt.% of D-230 replacement, resulted in a 25% increase in tensile strength, while the strain at break was retained compared to the control (pristine DGEBA/D-230 epoxy polymer). Similarly, the addition of lignin resulted in a substantial increase in stiffness (Young’s Modulus), as expected due to the lignin 3-dimensional aromatic structure that may facilitate enhanced π-π interactions with the bulk epoxy matrix [36,108,109,110]. When KL was used at higher contents and replaced a higher amount of D-230, an increased brittleness could be observed. It can be noted that the lower KL content induced better tensile strength and strain to failure performance. Nonetheless, the performance improvement of the composite with 5 wt.% of commercial D-230 replaced by lignin is indeed appreciable considering the glassy, rigid nature of the pristine epoxy polymer. Thus, KL has potential as a partial replacement for the commercial Jeffamine D-230 curing agent.

The storage modulus (E′) represents the energy storage and elastic response of a material. The E′ of all composites ranged between 1500 and 2748 MPa at 30 °C (Table 4). Various factors can affect the storage modulus E′, like crosslinking density, chain stiffness, or flexibility. Stiff chains in a network lead to a higher modulus (and higher T_g_) with respect to the network containing flexible chains [111]. Structures with bonds like aliphatic C-O and C-C show flexibility compared to highly branched and aromatic structures that confer stiffness to the chain. This can explain the gradual increase in E′ with the increase in loading of KL, presented in Figure 10 and Table 4, since higher rigidity is gained due to the increased amount of lignin’s phenolic structure compared to the polyether network chains of the pristine composite. This also suggests that KL does in fact interact with the epoxy network, and thus a more rigid matrix is created with decreased mobility (Scheme S2).

Valuable information about the cured epoxy networks can be extracted from the tanD vs. temperature curves. The peak of tanD corresponds to the glass transition temperature (T_g_). Regarding the T_g_ of the KL-containing composites, the same trend as the storage modulus is observed. The T_g_ is increased overall compared to the neat pristine epoxy, due to the hindering of the backbone mobility and immobilization of the network chains from the interaction with lignin (i.e., via its hydroxyls and/or via π-π aromatic interactions) and formation of a rigid and branched interphase/network. Intermolecular interactions or secondary bonding can affect segmental rotations and increase the rigidity of the composite and, consequently, the T_g_. In addition, an increasing amount of lignin results in the broadening of the glass transition due to some system inhomogeneity, as expected due to the higher polydispersity of lignin compared to diamine D-230 [34].

#### 3.2.4. Lignins as Additives in Lignin–Epoxy Composites—Thermo-Mechanical Properties

- Kraft Lignin as an Additive

**Figure 11:**
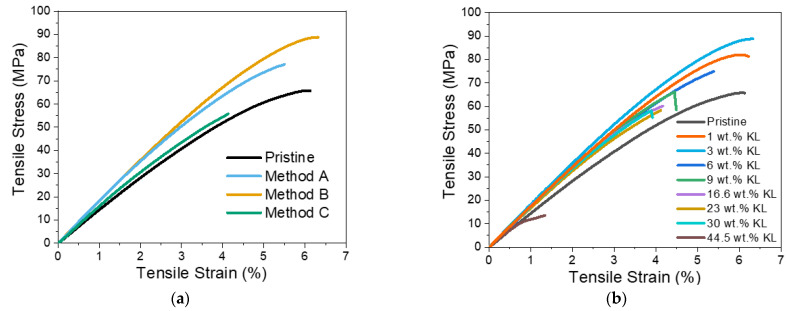
Tensile strength of KL–epoxy composites. (**a**) Effect of the preparation methods. (**b**) Effect of KL loading.

Blending of lignin with various polymers has been previously proven very successful [112]. Lignin, on its own, is usually immiscible with most apolar polymers due to its relatively polar nature. In this context, the pristine kraft lignin was evaluated as an additive, aiming at increasing the total mass of the prepared composite, or, in other words, replacing part of the DGEBA/D-230 mixture as a whole. In more detail, the stoichiometric molar ratio of commercial epoxy resin (DGEBA) and curing agent (Jeffamine D-230) was preserved and lignin was added in selected amounts. As can be seen from the tensile stress–strain data (Table 5 and Figure 11), lignin could be utilized and blended with the bulk epoxy polymer in various amounts (wt.%), and a greater mechanical improvement compared to the use of lignin as a reactive curing agent could be achieved.

As can be seen in Figure 11a, both simple stirring as well as sonication (without the use of EtOH) offered the best improvement in tensile strength, with the strain at break being unaffected.

With regard to the effect of KL loading (1 −44.5 wt.%), increases of 3.1%, 35.2% and 28.9% in tensile stress, strain, and stiffness were determined for the 3 wt.% KL-composites (Figure 11b and Table 5). Increasing the lignin content up to 9 wt.% resulted in a slight loss of tensile strain, whilst the strength and stiffness were still higher than the control pristine polymer (Table 5). A further increase in the KL content (16–44.5 wt.%) resulted in inferior properties, probably due to the bulk structure of lignin that creates defects in the dense crosslinked epoxy matrix. In addition, π-π stacking between lignin aromatic rings and other weak interactions like hydrogen bonding and van der Waals attractions between polymer chains may cause lignin aggregation that possibly result in impaired properties of the composites at such high loadings [23].

**Figure 12:**
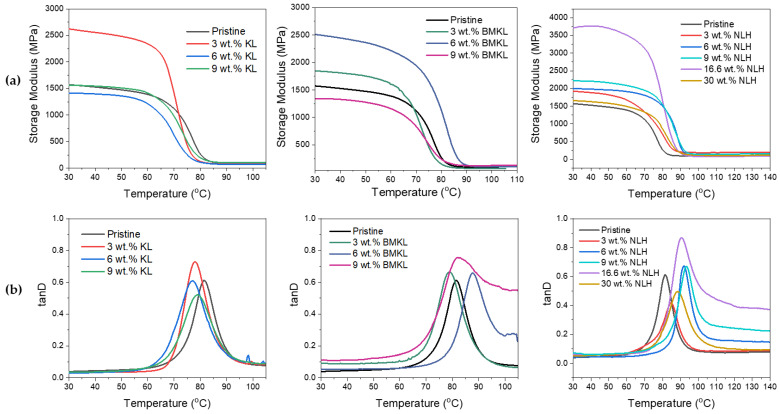
(**a**) Storage modulus and (**b**) tanD vs. temperature curves of initial (KL) and modified (BMKL and NLH) lignin–epoxy composites.

The storage modulus values at 30 °C and the glass transition temperatures (T_g_) of the KL–epoxy composites are given in Table 5. The neat pristine epoxy polymer has a higher T_g_ compared to all the KL containing samples. Epoxy resins are highly crosslinked materials; thus, the incorporation of a bulky, highly branched material like lignin into the epoxy network may possibly create disorder and decrease molecular chain packing, leading to a reduced T_g_ [113,114], as also explained above. The reduction of the T_g_ may also be attributed to the plasticizing effect [115] induced by the addition of lignin. Regarding the storage modulus E′, despite the substantially increased E′ exhibited by the composites containing 3 wt.% KL, the E′ of composites with higher loading did not vary significantly compared to the pristine polymer (Figure 12). In the case of KL as a crosslinker (Section 3.2.3), the addition of KL resulted in a higher E′ (Figure 10) due to the suggested formation of direct chemical bonds between lignin’s hydroxyls and epoxy rings (Scheme 1). This is not the case for the composites where KL was used as an additive, where, as discussed above, high lignin loadings may create agglomerates that disturb the dense highly crosslinked (due to the short chain aliphatic diamine curing agent) epoxy matrix, resulting in large volumes of empty space and voids (Scheme S2) and therefore inhibiting any potential chemical interaction between lignin and the epoxy polymer chains [114].

- Modified Lignins as Additives

**Figure 13:**
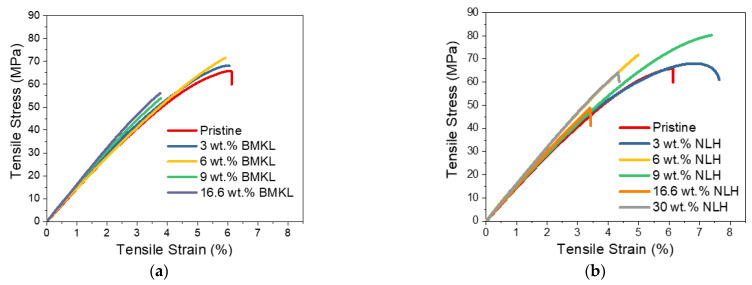
Stress–strain curves of (**a**) BMKL- and (**b**) NLH-epoxy composites.

The utilization of modified lignins (BMKL and NLH) as additives in epoxy composites was also investigated. Their mechanical and thermo-mechanical performances are presented in Figure 12 and Figure 13 and listed in Table 5.

The effect of lignin loading (ranging from 3 wt.% up to 30 wt.%), in the thermo-mechanical properties of the composites was evaluated by tensile stress and DMA analysis. As observed in the stress–strain curves (Figure 13a), the mechanical properties of the BMKL-containing composites are slightly increased compared to the pristine polymer, even when the BMKL content is as high as 6 wt.%. At higher loadings (9 and 16.6 wt.%), stress and strain are both reduced by almost half. Notably, increasing the BMKL content resulted in an increase in Young’s modulus (E), since lignin can offer stiffness to the composites.

The E′ curves of the lignin composites as a function of temperature are shown in Figure 12a. Overall, E′ was substantially increased with lignin incorporation. The T_g_ of the composites did not vary significantly by increasing the lignin loading, but the differentiation became higher for 6 wt.% BMKL loading (Figure 12b). Additionally, the composite with 9 wt.% BMKL displayed a wider tanD area, suggesting a possibly more inhomogeneous structure due to the high loading.

The stress–strain curves of the nanocomposites with nano-lignin (NLH) are shown in Figure 13b. The effect of lignin content is more pronounced compared to the BMKL-based composites. As shown, the dispersion of NLH particles in the epoxy network/matrix is ideal since their incorporation results in fully transparent composites. Indeed, the mechanical and thermo-mechanical properties of those composites are improved. The stress and strain values, as well as the stiffness, are improved, even up to 9 wt.% NLH loading, while 30 wt.% NLH loading results in retained strength with only a slight loss of strain compared to the pristine polymer. Additionally, the storage modulus is increasing substantially with the NLH content, even at a 30 wt.% content. The same increase is observed for the T_g_, which shows an increase of up to ca. 12 °C (Figure 12b). Furthermore, the tanD peak areas of all composites exhibit narrow distributions, confirming the formation of a homogeneous network.

The viscoelastic behavior presented thus far can be additionally correlated with the lignin particle size. As is visible in Figure 12a,b, the T_g_ as well as the E′ are influenced by lignin’s particle size. The smaller the particles, the higher the loading level, which can generate an improved storage modulus and T_g_, since smaller lignin particles reinforce the composites more efficiently. Reinforcement with KL (~67 μm) results in an increased E′ at only 3 wt.% loading. When BMKL lignin (~10 μm) is incorporated, E′ is increasing up to 6 wt.% loading. Finally, in the case of NLH, E′ is higher than the control sample, even up to 16.6 and 30 wt.% loading due to its nano-scale particle size (220 nm). A similar behavior can be observed with regard to the T_g_. In the case of KL-containing composites, the T_g_ was maintained at slightly lower values compared to the pristine polymer. The T_g_ slightly increased when BMKL was incorporated and was increased even further when NLH was added. In conclusion, the epoxy chain/network packing is improved as the nano- and micro-sized lignin particles are introduced in the epoxy matrix, contrary to the ineffective packing (and topographies of increased free volume) caused by KL’s irregular and bigger particles.

#### 3.2.5. Glycidylized Lignin as a DGEBA Replacement—Thermo-Mechanical Properties

Epoxy functionalities were introduced in kraft lignin structure after the reaction with epichlorohydrin. Glycidylized kraft lignin (GKL) was investigated as a bio-based epoxy prepolymer, aiming towards achieving the highest replacement of the BPA-based epoxy prepolymer DGEBA. To this end, the epoxy prepolymer EPON 828 was initially mixed with GKL at selected ratios so that the amount of GKL in the epoxy composite ranged between 3 and 50 wt.%. The amount of curing agent (Jeffamine D-230) was tuned at a stoichiometric ratio, maintaining a [1:1] ratio of amine reactive hydrogens (-NH) to epoxy rings (–C-O-C) deriving from both GKL and EPON 828.

**Figure 14:**
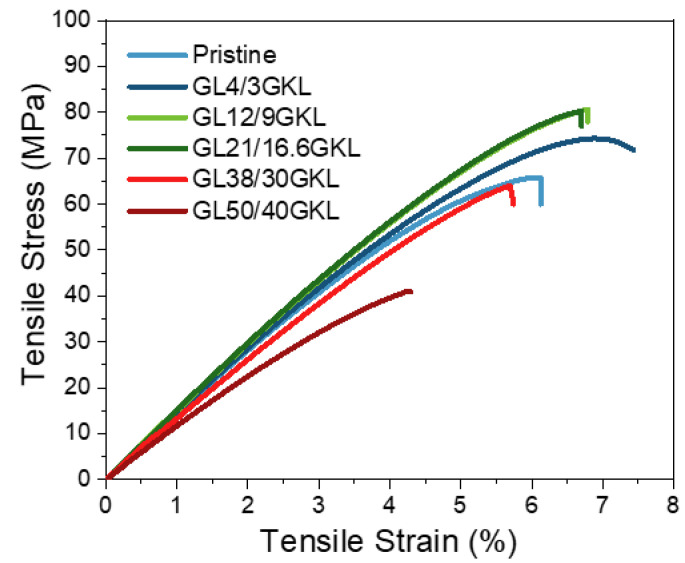
Stress–strain curves of glycidylized lignin GKL–epoxy composites after the replacement of 4 wt.%–50 wt.% of DGEBA with GKL.

**Figure 15:**
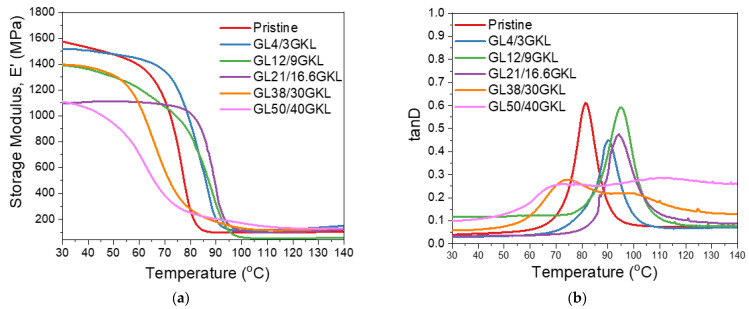
(**a**) Storage modulus and (**b**) tanD vs. temperature curves of glycidylized lignin GKL–epoxy composites, with GKL lignin as the DGEBA replacement in various percentages.

In most studies with lignin-based epoxy resins, the epoxy/diamine systems used are polyoxypropylene diamines (Jeffamine D-400 and D2000), isophorone diamine (IPD), or diethylenetriamine (DETA) [32]. Gioia et al. reported mixing issues regarding the preparation of glycidylized lignins and low(er) molar mass polyetheramine (i.e., Jeffamine D-400) formulations [33]. In the present work, we tried to test the potential utilization of glycidylized lignin with short-chain diamine, i.e., Jeffamine D-230. A good affinity between GKL and DGEBA and D-230 can be observed, as complete dissolution is achieved, generating transparent composites (Figure 9). The stress–strain curves of the GKL–epoxy composites are presented in Figure 14, their viscoelastic behavior is shown in Figure 15, and the values obtained are listed in Table 6. Substantial increases in stress, strain and stiffness are observed compared to the pristine polymer (control), even when high percentages of DGEBA are replaced by GKL (16.6 wt.% GKL content, which corresponds to 21 wt.% DGEBA replacement). Additionally, when 38 wt.% of DGEBA was substituted by GKL lignin (30 wt.% GKL content in the composite), the properties of the composite were retained. Regarding Young’s modulus (E) and the stiffness of the composites, by increasing the lignin content, the composites became more rigid (increase of E) up to the threshold of 16.6 wt.% GKL content. As seen in Figure 14, the tensile strength profiles of the 9 and 16.6 wt.% GKL composites are very similar, suggesting a GKL content–mechanical properties threshold. Beyond this point (≥ 30 wt.% GKL), the properties decline, although they are still similar to the pristine.

Regarding the viscoelastic behavior, by increasing the lignin content from 0 to 16.6 wt.%, a clear increase in the T_g_ can be observed (Figure 15 and Table 6). Initially, a 3 wt.% loading in the epoxy polymer caused an increase in the T_g_ by ~9 °C (from 81.5 °C to 90.1 °C), while higher lignin incorporation (16.6 wt.%) resulted in an overall increase of ~13 °C (94.1 °C). This behavior can be attributed to the ability of the GKL to not only catalyze the crosslinking by its free hydroxyls [36,115] but also participate in the crosslinking via its epoxide groups, thus affecting/increasing the crosslinking density [35]. A further increase in the lignin content up to 30 wt.% resulted in the broadening of the tanD peaks and the formation of a second peak. Despite the initial homogeneous dispersion, at this loading level, the insurgence of a second, GKL-rich phase is promoted. This phase shows different characteristics than the DGEBA-rich phase, thus the two T_g_s. Additionally, a decrease in the first T_g_ value is observed, which can be attributed to lower crosslinking density that is in turn caused by the high GKL content that increased the free volume [33,116].

The storage modulus E′ in the glassy state (30 °C) did not significantly vary after a 3% GKL incorporation with respect to the pristine polymer, while higher loading resulted in a lowering of E′. This can be attributed to the increased deformation of the dense epoxy polymer matrix at such high lignin loadings, as discussed above for the NHL and BMKL lignins, despite the increased bi-functionality of GKL via its remaining free hydroxyls and mostly via its epoxide groups. Overall, GKL can strengthen the DGEBA/D-230 skeletal structure through its chemical interaction, as well as improve its strength and toughness whilst achieving a significant amount of bio-based content in the epoxy resin.

#### 3.2.6. Thermal Properties of Lignin–Epoxy Composites

**Figure 16:**
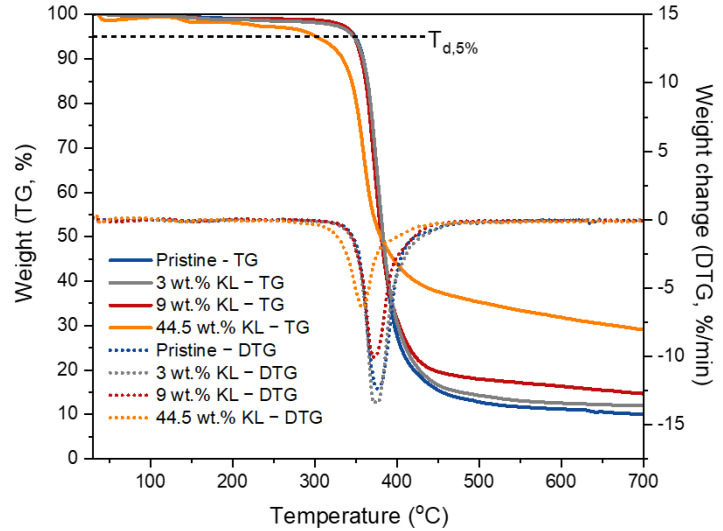
TGA/DTG curves of KL–epoxy composites showing the effect of KL loading.

The effect of KL on the thermal properties of the lignin–epoxy composites was investigated by TGA (Figure 16 and Table 7). Both pristine and lignin–epoxy composites exhibited one-step degradation. The addition of KL up to 9 wt.% did not impact the thermal stability of the epoxy polymer. On the other hand, when higher amounts of KL (44.5 wt.%) were incorporated, the thermal stability was reduced. The T_d,5%_ decreased to 301 °C, while the maximum degradation temperature (DTG_max_) was shifted to a lower temperature (ca. 18 °C lower than the control pristine polymer). The decrease in the thermal stability of the composites at high KL loadings may suggest a lower crosslinking density, as also discussed in the thermo-mechanical properties, thus requiring less energy to decompose [117,118]. In addition, higher KL loadings correspond to a higher amount of methoxy groups that, as established by Harvey et al. [119], can negatively affect the thermal stability of cured epoxies due to increased electron donation from the methoxy groups to the aromatic rings of the polymer. Interestingly, the residual mass at 700 °C (Char_700_) was significantly enhanced (from 9.9% up to 29.1%) with increasing lignin content [120], indicating that lignin can act as a char promoter, influencing the pyrolysis mechanisms of cured epoxides [47]. Additionally, it can be attributed to the reaction of lignin with itself (polycondensation), resulting in the formation of a condensed char-like material when heated under nitrogen during the TGA measurement. Notably, the lignin-containing composites showed intumescent properties, as foaming char residue was observed after the TGA analysis (Figure S7). The formation of foaming char (intumescent layer) testifies the potential utilization of lignin as a fire retardant additive in epoxy polymers, as well as other polymer types.

**Figure 17:**
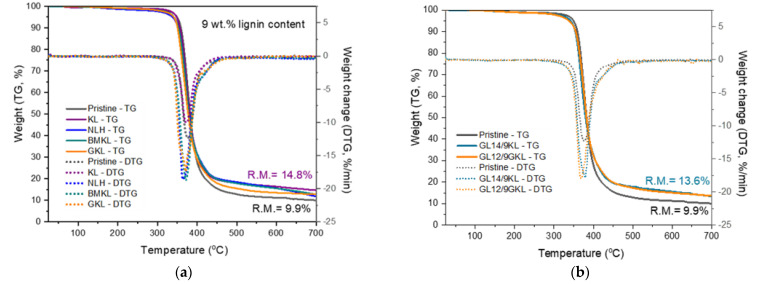
TGA/DTG curves of lignin-containing composites. (**a**) Effect of a 9 wt.% content of various lignins in composites, and (**b**) effect of D230 and DGEBA replacement by 9 wt.% KL and GKL, respectively.

As to the effect of various lignin types on the epoxy composites, the incorporation of 9 wt.% of BMKL, NLH, and GKL did not affect either the thermal stability or the degradation temperature (Figure 17 and Table 7). The residual mass (Char_700_) observed at 700 °C was increased and presented foaming char, as observed in the KL–epoxy composites, confirming the huge potential towards the development of thermally stable and flame-retardant materials.

#### 3.2.7. Antioxidant Properties and Solvent Resistance of Lignin–Epoxy Composites

**Figure 18:**
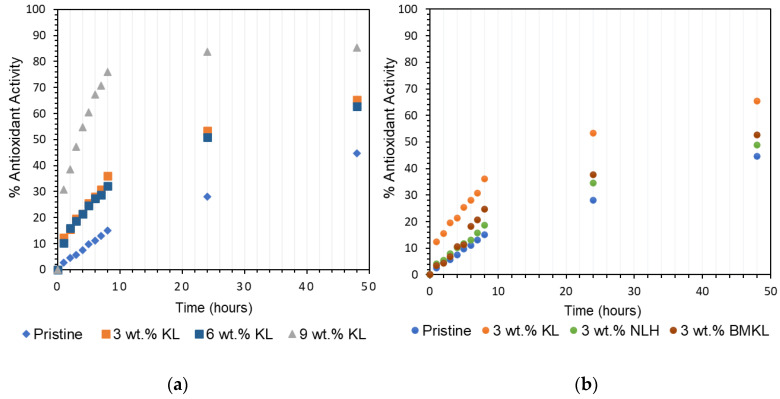
Antioxidant activity of (**a**) KL lignin in epoxy composites at various contents (3–9 wt.%) and (**b**) various lignins at a 3 wt.% content in epoxy composites.

**Figure 19:**
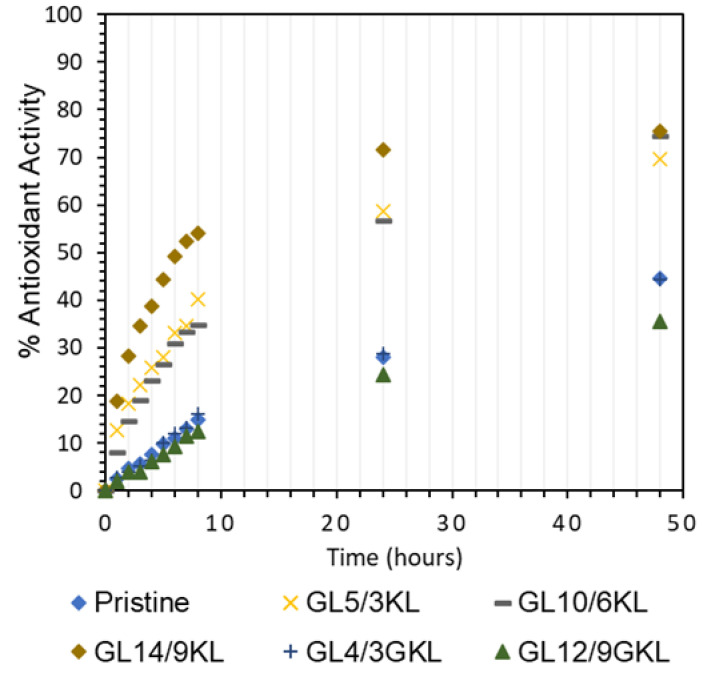
Antioxidant activity of KL lignin vs. GKL lignin in epoxy composites.

The radical scavenging activity of lignin–epoxy composites was investigated using the DPPH assay method, and the results are shown in Figure 18 and Figure 19. Lignin is a well-known radical scavenger due to the high concentration of phenolic Ohs [121,122,123] and thus has been utilized in many applications [36,122,124,125].

**Figure 20:**
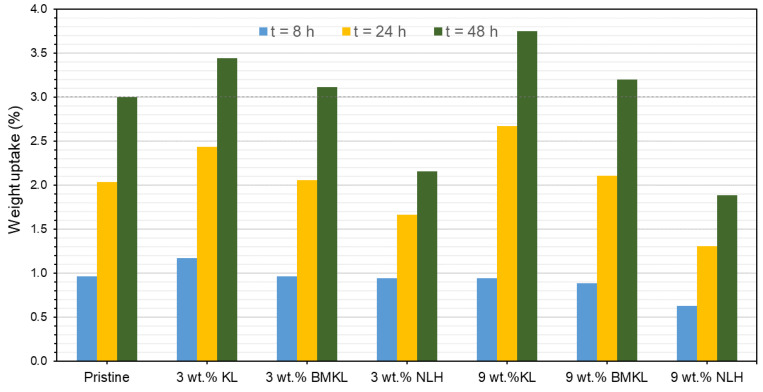
Solvent (EtOH) resistance (expressed as weight uptake) of the pristine epoxy polymer and the lignin–epoxy composites with various contents (3% relative standard deviation).

Overall, the presence of lignin (KL, BMKL, and NLH) in the epoxy composites resulted in substantial scavenging activity of free radicals as compared to the pristine polymer. As expected, the higher the lignin content, the higher the antioxidant activity (Figure 18a). KL–epoxy composites exhibit significantly higher scavenging activity compared to BMKL– and NLH–epoxy composites (Figure 18b). This can be associated with lignins’ particle size and the polymeric chain packing achieved after lignin incorporation. As discussed above, the reduction in the lignin particle size influenced the packing and crosslinking of the composites, as smaller sized lignin enhanced the crosslinking degree. It should be pointed out that in order to measure the antioxidant activity, the composites need to be submerged in a DPPH-containing EtOH solution. Composites with a less compacted crosslinking network, i.e., KL–epoxy composites, are more susceptible to solvent diffusion (Scheme S2), and thus display a higher DPPH radical scavenging ability. Solvent resistance tests have been carried out to confirm this hypothesis. The results (Figure 20 and Figure S8) highlight that KL-containing composites exhibited a higher weight uptake upon EtOH absorption compared to the respective composites with BMKL and NLH. Overall, the nano-lignin NLH–epoxy composites showed the lowest EtOH uptake compared to the pristine epoxy resin.

Last, but not least, the partial replacement of D-230 by KL (GL*x*/*y*KL) did not influence the antioxidant capacity (Figure 19), i.e., provided a similar substantial improvement, compared to the composites where KL was used as an additive (Figure 18a). On the other hand, as expected, the addition of GKL lignin (GL*x*/*y*GKL) does not provide any additional radical scavenging ability with respect to the control (Figure 19), since most of GKL’s phenolic hydroxyls have been utilized to graft the epoxide rings.

## 4. Conclusions

Lignin–epoxy composites were successfully synthesized from kraft lignin (KL) incorporated in DGEBA and D-230 glassy epoxy systems. KL was utilized for the partial replacement of the curing agent and the DGEBA prepolymer (after glycidylation) or as a reactive additive. Additionally, KL lignin was ball milled (BMKL) and ultrasonicated (NLH) to study the effect of the lignin particle size reduction to the few micrometers/nano-size level. Overall, it was demonstrated that the pristine KL is reactive towards DGEBA’s epoxy ring opening via its functional hydroxyls. Thus, an up to 14 wt.% replacement of D-230 curing agent was effectively applied, leading to composites with similar/enhanced properties. When pristine KL was used as an additive, i.e., by replacing certain parts of the whole DGEBA/D-230 mixture/polymer, an up to 30 wt.% lignin loading was accomplished without compromising the thermo-mechanical properties, while at the same time offering significant improvement of the antioxidant properties.

A lignin particle size reduction to ca. 10 μm in the BMKL sample (from 67 μm of the pristine kraft lignin) and to ca. 220 nm in the NHL sample resulted in better dispersion and completely transparent samples with nano-lignin, while the thermo-mechanical properties were further improved with up to ca. 30 wt.% nano-lignin compared to the use of the pristine (unmodified) lignin. The glycidylized lignin (GKL) offers even more improvements to dispersion and thermo-mechanical properties owing to its dual functionality, i.e., by its grafted epoxide rings and by the remaining (after glycidylation) hydroxyl and carboxyl groups. A DGEBA replacement of up to 38 wt.% was demonstrated, leading to lignin–epoxy composites with properties that allow their utilization in highly demanding applications in which classical phenolic epoxy polymers are being used.

In addition to the sustained/improved mechanical, thermal and antioxidant properties, the nano-lignin–epoxy composites offer higher resistance to solvent (EtOH) uptake, while all lignins tested exhibit the potential to be utilized as fire retardant additives in epoxy polymers, as well as other polymer types, due to their char formation capability.

## Figures and Tables

**Table 1:** Physicochemical characteristics of initial kraft and modified lignins as obtained from GPC, N_2_ porosimetry, and elemental analysis.

	GPC			N_2_ Porosimetry		Elemental Analysis				
Lignin	Mw (g/mol)	Mn (g/mol)	PDI	Total Pore Volume (cc/g)	Surface Area (m^2^/g)	N (wt.%)	C (wt.%)	H (wt.%)	S (wt.%)	O (wt.%) *
Kraft	6500	1400	4.7	0.009	0.8	1.44	61.13	5.73	1.49	30.21
BMKL	3400	1000	3.4	0.042	5.1	2.97	62.67	5.29	1.27	27.80
NLH	3200	650	4.9	0.104	8.6	3.34	51.64	4.75	2.92	37.35
GKL	5400	1500	3.6	0.013	1.7	2.82	62.63	5.72	1.11	27.72

* Calculated by difference.

**Table 2:** Thermal properties of the various lignin samples, as obtained by DSC and TGA analyses.

Lignin	T_g_ (°C) ^a^	T_d,5%_ (°C) ^b^	DTG_max_ (°C) ^b^	Residue at 800 °C (%) ^b^
KL	143	219	372	40.7
BMKL	148	220	343	40.1
NLH	116	233	344	47.2
GKL	103	294	325 & 403	46.4

^a^ Determined from DSC; ^b^ determined from TGA.

**Table 3:** Quantitative assignments of interunit linkages, structural units’ abundances, and functional group (OH and -COOH) contents of kraft and treated lignins, as determined by 2D-HSQC and ^31^P-NMR spectroscopy.

	KL	BMKL	NLH	GKL	
**Lignin interunit bonds—Abundance (per 100 Ar ^a^)**					**Chemical shifts (δ_C_/δ_H_)**
Arylglycerol-β-aryl ethers (β-O-4)	22.8	23.7	18.3	5.7	71.1/4.8
Pinoresinols (β-β)	10.9	12.3	4.8	4.1	84.8/4.6
Phenyl coumaran (β-5)	6.9	7.6	4.5	2.7	86.9/5.5
Diarylpropane (β-1)	1.4	3.9	3.9	3.4	48.7/3.1
**Lignin aromatic units—Abundance (%)**					
**G/H ratio**	91.2/8.8	91.7/8.3	92.3/7.7	96.5/3.5	
**Lignin functional groups—Abundance (mmol/gr) ^b^**					**Chemical shifts (δ_31*p*_)**
Aliphatic OH	2.25	2.30	2.47	2.43	145–150
Condensed OH	1.59	1.49	1.05	0.04	140.3–144.5
Non-condensed OH	2.02	2.13	2.58	0	137.2–140.3
Guaiacyl OH	1.81	1.83	2.31	0	138.4–140.3
*p*-Hydroxyphenyl OH	0.21	0.30	0.27	0	137.2–138.4
Total PhOH	3.61	3.62	3.63	0.04	137.2–144.3
-COOH	0.45	0.46	0.94	0	134–136
Epoxy content ^c^	-	-	-	3.80	-

^a^ Ar = aromatic units, ^b^ from ^31^P-NMR, ^c^ from ^1^H-NMR.

**Table 4:** Mechanical and thermo-mechanical properties of kraft lignin–epoxy composites.

		Tensile Strength			DMA	
KL Content	D-230 Replacement	Ultimate Strength	Strain at Break	Young Modulus, E	Storage Modulus E′ at the Glassy State ^1^	T_g_ ^2^
(wt.%)	(wt.%)	(MPa)	(%)	(MPa)	(MPa)	(°C)
0	0	65.6 ± 3.6	6.1 ± 0.5	1329 ± 22	1580 ± 74	81.5 ± 0.6
3	5	81.6 ± 5.6	6.0 ± 0.6	1686 ± 15	1500 ± 62	86.5 ± 1.7
6	10	55.6 ± 4.7	3.7 ± 0.4	1545 ± 118	2082 ± 130	86.1 ± 1.1
9	14	45.2 ± 0.9	2.8 ± 0.1	1688 ± 46	2748 ± 210	86.3 ± 2.4
45	100	Sample too brittle for tensile and DMA measurement				

^1^ The storage modulus was determined at 30 °C; ^2^ Tg was determined by the peak of the tanD vs. temperature curve from DMA.

**Table 5:** Mechanical and thermo-mechanical properties of lignin–epoxy composites.

	Tensile Strength			DMA	
KL Content	Ultimate Strength	Strain at Break	Young’s Modulus, E	Storage Modulus E′ at the Glassy State ^1^	T_g_ ^2^
(wt.%)	(MPa)	(%)	(MPa)	(MPa)	(°C)
Pristine	65.6 ± 3.6	6.1 ± 0.5	1329 ± 22	1580 ± 74	81.5 ± 0.6
1 wt.% KL	82.0 ± 5.3	6.2 ± 0.5	1644 ± 53	2512 ± 180	74.8 ± 0.9
3 wt.% KL	89.1 ± 5.7	6.3 ± 0.4	1714 ± 38	2621 ± 322	78.0 ± 1.6
6 wt.% KL	75.0 ± 1.4	5.4 ± 0.2	1613 ± 14	1411 ± 152	77.1 ± 1.2
9 wt.% KL	66.3 ± 1.2	4.5 ± 0.2	1656 ± 36	1561 ± 181	79.1 ± 2.2
16.6 wt.% KL	60.2 ± 1.8	4.2 ± 0.1	1620 ± 26	1515 ± 71	92.8 ± 1.5
23 wt.% KL	58.3 ± 0.8	4.1 ± 0.3	1550 ±69	-	-
30 wt.% KL	58.2 ± 4.1	3.9 ± 0.4	1598 ± 39	2691 ± 69	60.9 ± 2.1
44.5 wt.% KL	13.6 ± 10.5	1.4 ± 2.1	800 ± 287	-	-
3 wt.% BMKL	68.4 ± 1.4	6.1 ± 0.3	1400 ± 55	1847 ± 95	78.9 ± 0.7
6 wt.% BMKL	71.6 ± 4.3	5.9 ± 0.4	1329 ± 13	2513 ± 185	87.7 ± 2.1
9 wt.% BMKL	54.0 ± 1.7	3.8 ± 0.2	1536 ± 57	1341 ± 72	82.5 ± 1.5
16.6 wt.% BMKL	56.1 ± 6.3	3.8 ± 0.4	1569 ± 91	-	-
3 wt.% NLH	68.2 ± 1.1	7.6 ± 0.1	1379 ± 23	1928 ± 88	84.8 ± 1.2
6 wt.% NLH	72.0 ± 4.3	5.0 ± 0.2	1520 ± 41	2001 ± 90	91.8 ± 1.6
9 wt.% NLH	80.5 ± 6.1	7.4 ± 0.6	1361 ± 19	2229 ± 209	93.6 ± 1.1
16.6 wt.% NLH	48.7 ± 4.6	3.4 ± 0.2	1498 ± 53	3709 ± 422	90.7 ± 0.9
30 wt.% NLH	64.2 ± 5.6	4.3 ± 0.5	1543 ± 34	1662 ± 110	88.2 ± 1.9

^1^ The storage modulus was determined at 30 °C. ^2^ T_g_ was determined from the peak of the tanD vs. temperature curve using DMA.

**Table 6:** Mechanical and thermo-mechanical properties of glycidylized lignin–epoxy composites.

	Tensile Strength			DMA	
KL Content	Ultimate Strength	Strain at Break	Young’s Modulus, E	Storage Modulus at the Glassy State ^1^	T_g_ ^2^
(wt.%)	(MPa)	(%)	(MPa)	(MPa)	(°C)
Pristine	65.6 ± 3.6	6.1 ± 0.5	1329 ± 22	1580 ± 74	81.5 ± 0.6
GL4/3GKL	74.4 ± 2.2	7.5 ± 0.1	1362 ± 54	1516 ± 95	90.1 ± 1.7
GL12/9GKL	80.8 ± 1.5	6.8 ± 0.3	1401 ± 17	1390 ± 45	95.1 ± 2.3
GL21/16.6GKL	80.4 ± 1.1	6.7 ± 0.3	1415 ± 5	1095 ± 130	94.1 ± 1.4
GL38/30GKL	64.1 ± 3.3	5.7 ± 0.4	1241 ± 69	1397 ± 320	74.3 & 98.9
GL50/40GKL	41.3 ± 32	4.3 ± 0.4	1052 ± 10	1109 ± 51	70.5 & 109

^1^ The storage modulus was determined at 30 °C. ^2^ T_g_ was determined from the peak of the tanD vs. temperature curve using DMA.

**Table 7:** Thermal properties of kraft lignin (KL)-containing composites.

	Sample	T_d,5%_ (°C)	DTG_max_ (°C)	Char_700_ (%)
**Effect of the** **kraft content**	Pristine	348	376	9.9
	3 wt.% KL	348	374	11.8
	9 wt.% KL	348	374	14.8
	44.5 wt.% KL	301	358	29.1
**Effects of the** **lignin type andcontent**	3 wt.% BMKL	348	378	14.6
	9 wt.% BMKL	341	372	12.6
	3 wt.% NLH	338	375	12.4
	9 wt.% NLH	337	365	11.9
	3 wt.% GKL	353	376	12.0
	9 wt.% GKL	341	371	12.9
**Effect of** **replacement** **by lignin**	GL14/9KL	341	379	13.6
	GL12/9GKL	336	368	13.5

## Data Availability

Data are contained within the article and supplementary material.

## Supplementary Materials

The following supporting information can be downloaded at https://www.mdpi.com/article/10.3390/polym16040553/s1: Figure S1. GPC curves of pristine and modified lignins; Figure S2. FT-IR-ATR spectra of pristine kraft and treated lignins; Figure S3. EDS maps of pristine and modified kraft lignins; Figure S4. ^31^P-NMR spectra of pristine and modified lignins; Scheme S1. Proposed mechanisms involved in the glycidylation reaction and self-crosslinking; Scheme S2. Illustration depicting the influence of “lignin as curing agent” vs. “lignin as additive” in the DGEBA/D-230 polymeric network. “Lignin as curing agent” interacts with the polymeric matrix, contrary to the “lignin as additive” that creates lignin clusters (large free volume) and decreased crosslinking; Table S1. NMR assignments of major structures in the 2D-HSQC spectra of Kraft lignins; Figure S5. Photographs capturing the preparation steps of NLH– and GKL–epoxy composites. A) Lignins dispersed in EtOH, B) suspension of swelled lignins after sonication (B), and total dispersion of lignins in the DGEBA/D-230 epoxy system; Figure S6. Optical microscopy images of transparent lignin–epoxy composites with 9, 16.6 and 30 wt.% NLH and GKL lignins; Figure S7. Photographs of lignin–epoxy composites exhibiting intumescent properties (foaming scar layer); Figure S8. Solvent resistance (expressed as weight uptake) as a function of time/days of the pristine epoxy polymer and the lignin–epoxy composites with various lignin contents using ethanol.
