# Supplementary material for: Kraft (Nano)Lignin as Reactive Additive in Epoxy Polymer Bio-Composites
Source: Polymers (Basel). 2024 Feb 18;16(4):553. doi: 10.3390/polym16040553 (PMC10893208; doi:10.3390/polym16040553)
Supplement: Supplementary file 1 [file polymers-16-00553-s001.zip › polymers-2821845-supplementary.pdf]

Supporting Information

Kraft (Nano)Lignin as Reactive Additive in Epoxy Polymer Bio-Composites

Christina P. Pappa [1], Simone Cailotto [2], Matteo Gigli [2], Claudia Crestini [2] and Konstantinos S. Triantafyllidis [1,3,*]

[1] Department of Chemistry, Aristotle University of Thessaloniki, 54124 Thessaloniki, Greece; christipappa@chem.auth.gr

[2] Department of Molecular Sciences and Nanosystems, Ca' Foscari University of Venice, 30170 Venice Mestre, Italy; claudia.crestini@unive.it (C.C.)

[3] Center for Interdisciplinary Research and Innovation (CIRI-AUTH), 57001 Thessaloniki, Greece

* Correspondence: Correspondence: ktrianta@chem.auth.gr

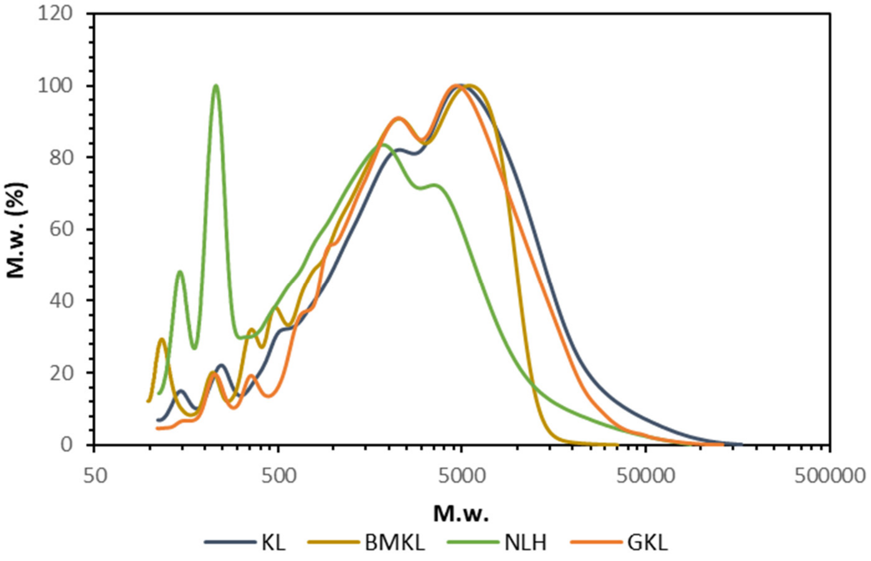

Figure S1. GPC curves of pristine and modified lignins

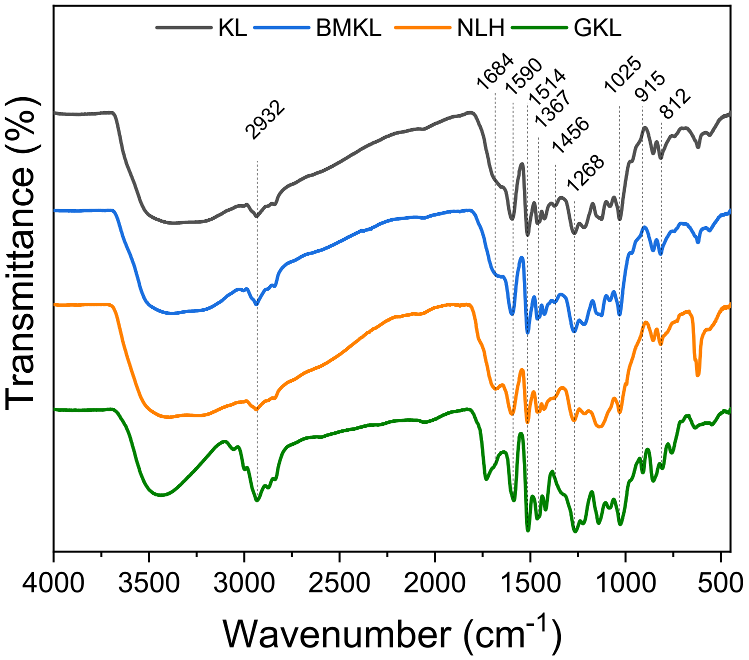

Figure S2. FT-IR-ATR spectra of initial kraft and treated lignins.

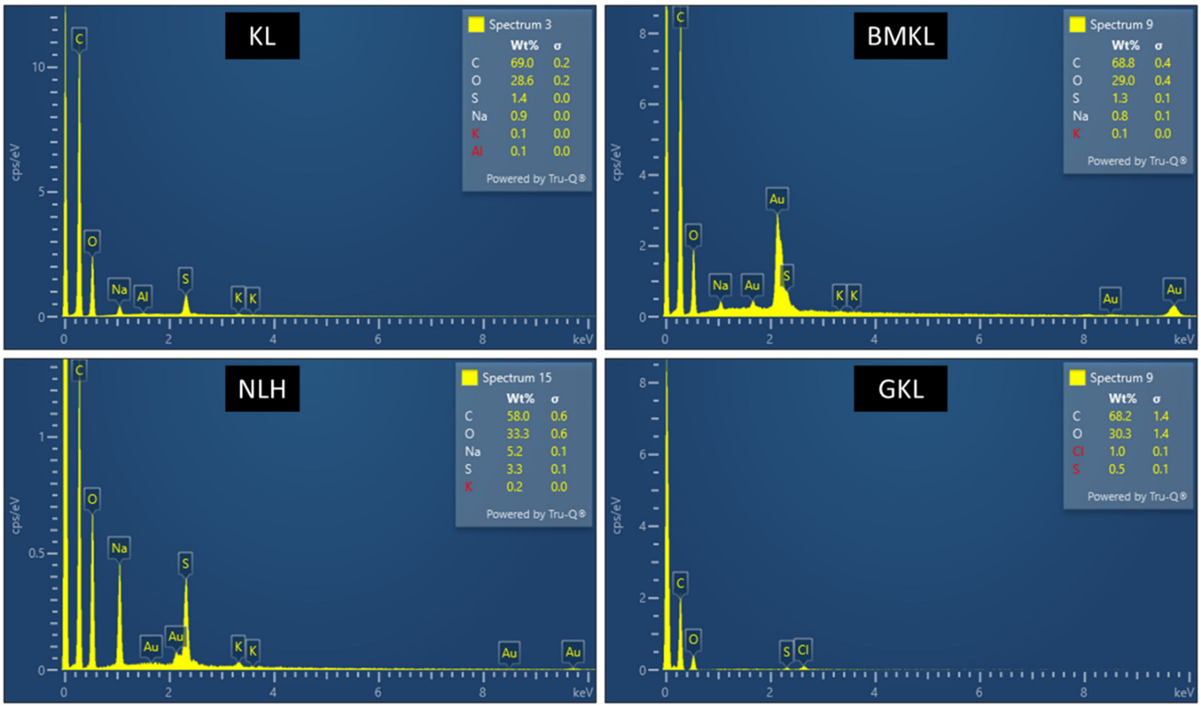

Figure S3. EDS maps of pristine and modified kraft lignins

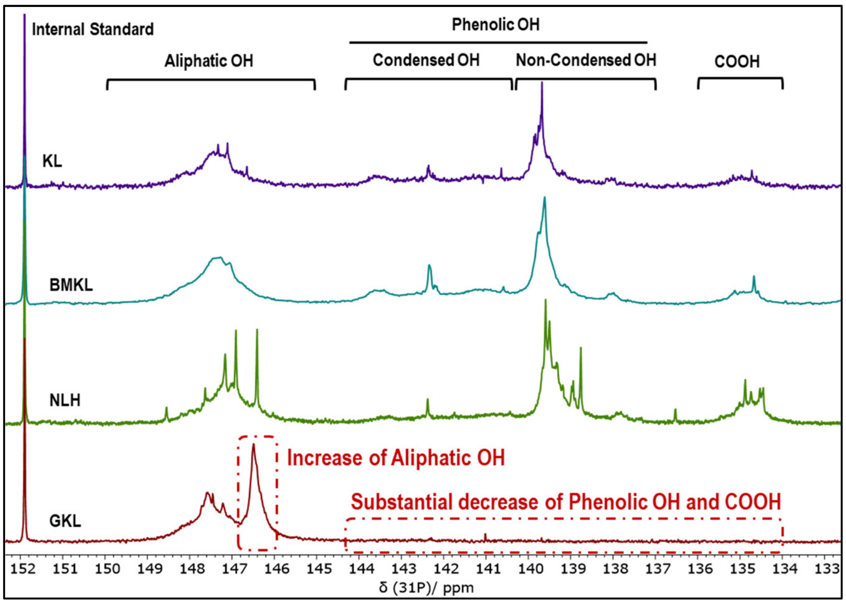

Figure S4. ^{31}P-NMR of pristine kraft and modified lignins

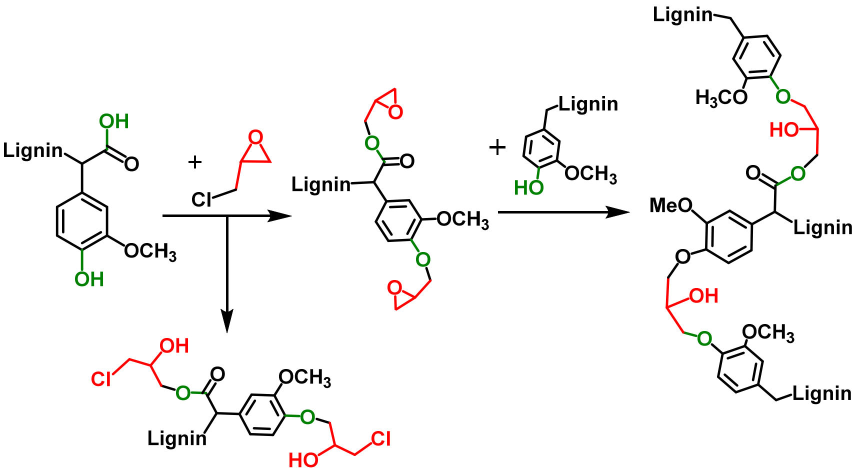

Scheme S1. Proposed mechanisms involved in the glycidylation reaction and self-crosslinking

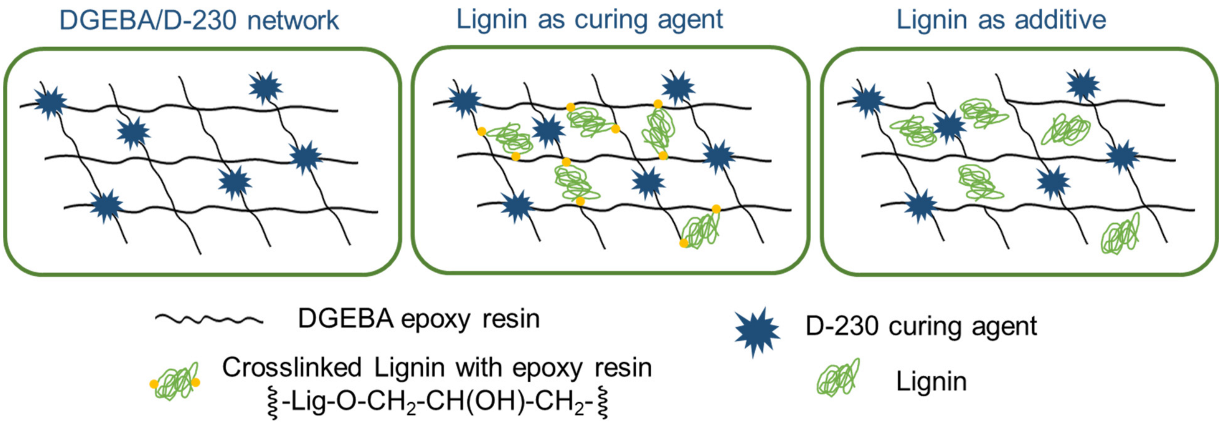

Scheme S2. Illustration of depicting the influence of "lignin as curing agent" vs "lignin as additive" in the DGEBA/D-230 polymeric network. "Lignin as curing agent" chemically interacts with the polymeric matrix via crosslinks, contrary to the "lignin as additive" that creates lignin clusters (large free volume) and decreased crosslinking but still being able to induce π-π interactions between the aromatic units of lignin and the aromatic backbone of DGEBA.

Table S1. NMR assignments of major structures in the 2D-HSQC spectra of Kraft lignins.

Label	δ_C/δ_H (ppm)	Assignment
**A_α (β-o-4)**	71.1/4.8	C_α-H_α in β-O-4 substructures (A)
**A_β**	81.3, 83.9/4.7, 4.3	C_β-H_β in β-O-4 substructures (A)
**A_γ**	59.9/3.3	C_γ-H_γ in β-O-4 substructures (A)
**B_α (β-β)**	84.8/4.6	C_α-H_α in β-β resinol (B)
**B_β**	53.3/3.04	C_β-H_β in resinol substructures (B)
**B_γ**	70.6/4.1	C_γ-H_γ in resinol substructures (B)
**C_α (β-5)**	86.9/5.5	C_α-H_α in phenylcoumaran substructures (C)
**C_β**	53.1/3.9	C_β-H_β in phenylcoumaran substructures (C)
**C_γ**	62.9/3.8	C_γ-H_γ in phenylcoumaran substructures (C)
**β-1**	48.7/3.1	C_β-H_β in diarylpropane substructures (β-1)
**G_2**	108.8-113.8/6.6-7.3	C_2-H_2 in guaiacyl units (G)
**G'_2**	109.4-113.9/7.3-7.6	C_2–H_2 in oxidized (C_α=O) guaiacyl units (G')
**G_5**	115.3/6.4-7.1	C_5-H_5 in guaiacyl units (G)
**G_6**	120.7/6.4-7.3	C_6-H_6 in guaiacyl units (G)
**G'_6**	123.3/7.5, 126.2/7.41, 125.8/7.25	C_6–H_6 in oxidized (C_α=O) guaiacyl units (G')
**$H_{2,6}$**	128.6/7.2	$C_{2,6}$–$H_{2,6}$ in p-hydroxyphenyl units (H)
**St**	125.8/7.0	C_β-H_β in stilbene (St)

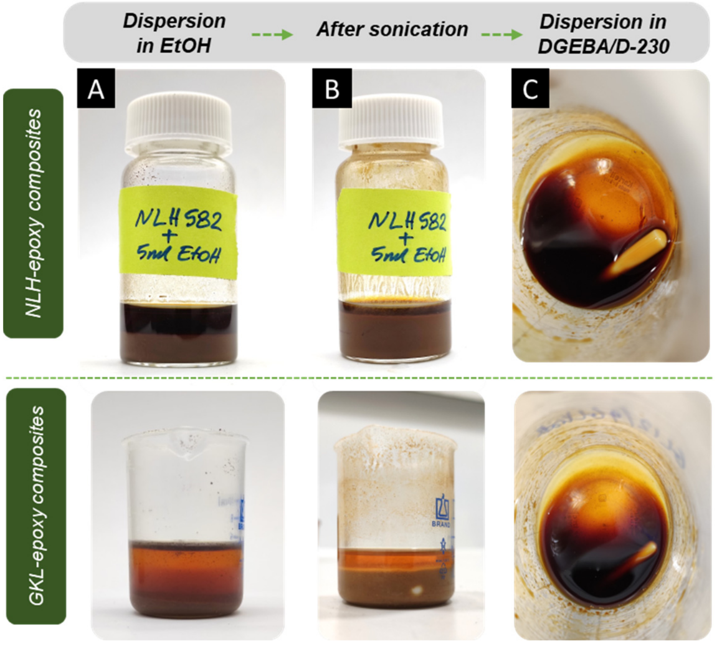

Figure S5. Photographs capturing the preparation steps of NLH- and GKL-epoxy composites. (A) Lignins dispersed in EtOH, (B) suspension of swelled lignins after sonication (C) dispersion of lignins in the final DGEBA/D-230 epoxy system

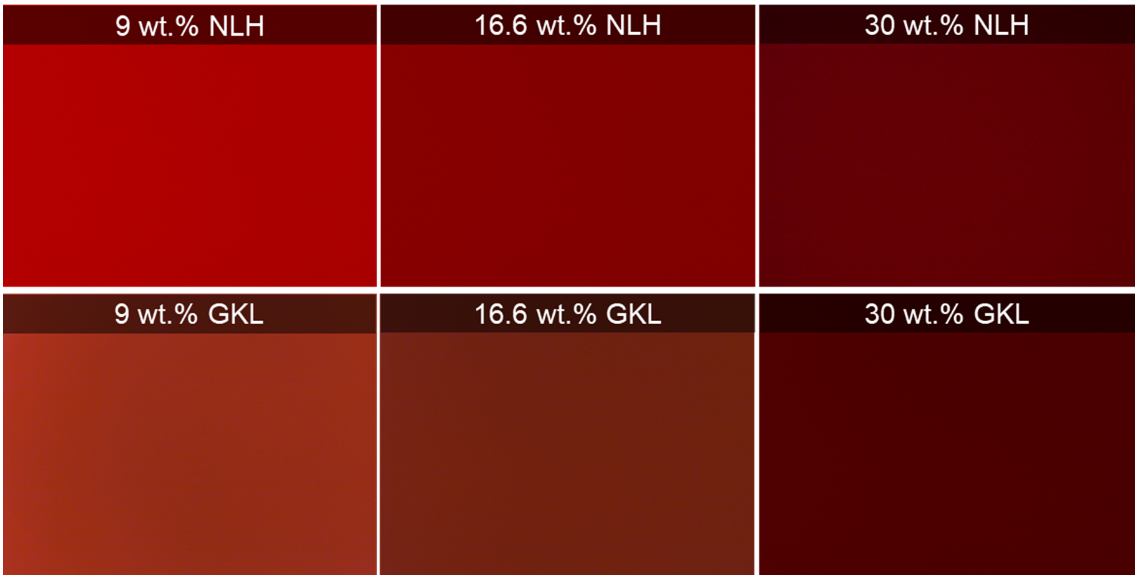

Figure S6. Optical microscopy images of transparent lignin-epoxy composites with 9, 16.6 and 30 wt.% NLH and GKL lignins.

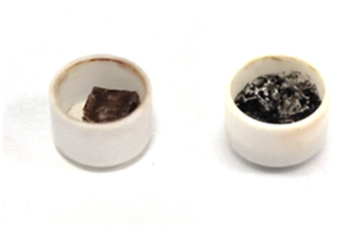

Figure S7. Photographs of lignin-epoxy composites exhibiting intumescent properties (foaming scar layer)

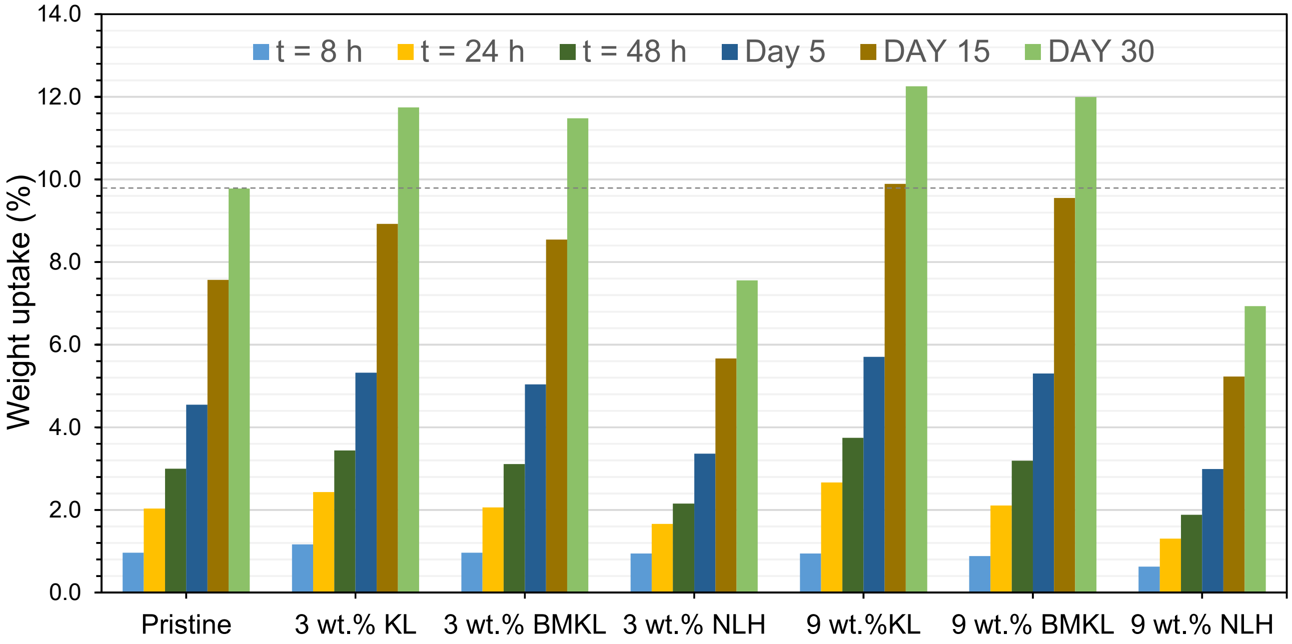

Figure S8. Solvent resistance (expressed as weight uptake) as a function of time/days of the pristine epoxy polymer and the lignin-epoxy composites at various lignin contents using ethanol (EtOH; 3% relative standard deviation) (Pristine polymer control indicated by dashed line)
